# Seizure classification with selected frequency bands and EEG montages: a Natural Language Processing approach

**DOI:** 10.1186/s40708-022-00159-3

**Published:** 2022-05-27

**Authors:** Ziwei Wang, Paolo Mengoni

**Affiliations:** 1 Institute of Interdisciplinary Studies, Hong Kong Baptist University, Kowloon Tong, Hong Kong SAR China; 2 Department of Journalism, Hong Kong Baptist University, Kowloon Tong, Hong Kong SAR China

**Keywords:** Seizure, Electroencephalography, Frequency bands selection, Natural Language Processing, Classification, Epileptic seizure

## Abstract

Individualized treatment is crucial for epileptic patients with different types of seizures. The differences among patients impact the drug choice as well as the surgery procedure. With the advance in machine learning, automatic seizure detection can ease the manual time-consuming and labor-intensive procedure for diagnose seizure in the clinical setting. In this paper, we present an electroencephalography (EEG) frequency bands (sub-bands) and montages selection (sub-zones) method for classifier training that exploits Natural Language Processing from individual patients’ clinical report. The proposed approach is targeting for individualized treatment. We integrated the prior knowledge from patient’s reports into the classifier-building process, mimicking the authentic thinking process of experienced neurologist’s when diagnosing seizure using EEG. The keywords from clinical documents are mapped to the EEG data in terms of frequency bands and scalp EEG electrodes. The data of experiments are from the Temple University Hospital EEG seizure corpus, and the dataset is divided based on each group of patients with same seizure type and same recording electrode references. The classifier includes Random Forest, Support Vector Machine and Multi-Layer Perceptron. The classification performance indicates that competitive results can be achieve with a small portion of EEG the data. Using the sub-zones selection for Generalized Seizures (GNSZ) on all three electrodes, data are reduced by nearly 50% while the performance metrics remain at the same level with the whole frequency and zones. Moreover, when selecting by sub-zones and sub-bands together for GNSZ with Linked Ears reference, the data range reduced to 0.3% of whole range, and the performance deviates less than 3% from the results with whole range of data. Results show that using proposed approach may lead to more efficient implementations of the seizure classifier to be executed on power-efficient devices for long lasting real-time seizures detection.

## Introduction

Epileptic seizure is one of the most common neurologic disorders that affects the population of all age groups worldwide [1]. Epilepsy is characterized by unprovoked and recurrent seizures and is manifested as a brain spectrum disorder [2]. Seizure is the temporal interruption of normal electrical brain function with burst alterations of neurologic regulation triggered by abnormal electrical neurons discharge [3]. Treatment of seizure includes medicines and brain surgeries, but medically intractable seizures that severely impact some patients’ quality of life still exist.

The diagnosis and monitoring of seizures can be analyzed with electroencephalography (EEG). EEG is neuro-electrophysiologic signals that represent the brain activities acquired from electrodes either implanted subdurally (intracranial EEG) or placed along the scalp (scalp EEG). Given the low-cost and non-invasive nature of scalp EEG, it is still a widely used tool for probing neural functions.

The gold standard to identify seizures is the visual recognition by a trained neurophysiologist using the EEG data where the abnormal electrical morphology is discovered. This manual procedure is labor-intensive as well as time-consuming in the clinical setting, it is subject to electrical signals interference by external noise and artifacts, and the subjective nature of such analysis can lead to disagreement among neurophysiologists.

Automated seizure detection from EEG recordings have been investigated by researchers since 1970s [4]. Models are built to distinguish patterns in brain signals that manifest of epileptic seizures. The models are framed in two typical steps: feature engineering and classification of the ictal/inter-ictal (during seizure/in-between seizures) signals. For a real-world seizure detection problem, the machine learning classification models need to be built with cost-consciousness trying to avoid the intermediate steps for feature computation that have high computational cost. The frequency domain features have proved to be more computationally efficient than time domain [5] and time–frequency domain features.

Background EEG frequency band $(\alpha , \beta , \theta , \delta , \gamma )$ oscillations have been intensively studied in brain normal function. These frequency bands can be recorded during state of wake or sleep: occipital alpha frequency activity $(\alpha )$ observed with a relax state while eyes are closed, frontal and central beta $(\beta )$ and gamma oscillations $(\gamma )$ during alert and vigilance mental state, theta frequency activity $(\theta )$ during sleep or memory tasks, and frontal delta rhythm $(\delta )$ that was recorded while sleep. Nevertheless, the seemingly normal EEG background bands contain clear abnormalities which have been shown as a significant prognostic tool [6–9].

The selection of EEG channels/montages is widely studied [10] for faster detection and noise removal. The EEG montages refers to the electrodes located on scalps connecting the patient and recording device. Since brain signal conduct in a non-linear and dynamic manner, the recorded electrical voltage is impacted by electrode locations significantly [11]. Neurologists select the channels using their prior knowledge and for an efficient approach it is vital to select the montages which carry the most discriminative information. It has been demonstrated that it is effective to select only a small number of montages for seizure detection [12, 13]. For computer scientists, the process of selecting montages requires additional steps of generation and evaluation of certain electrodes. The subset channels are generated from whole set using various statistical measures [14–16].

In this study, we use a natural language processing approach for the efficient selection of frequency bands (sub-bands) and scalp EEG electrodes (sub-zones). By consolidating each patient’s clinical report, we aim to integrate the medics’ prior knowledge into the classifier-building process. In particular, we classify seizure ictal/inter-ictal phases with sub-bands and sub-zones selection from six designed inputs. The three types of frequency band inputs are: the whole frequency range provided in data corpus, the background frequency EEG bands $(\alpha , \beta , \theta , \delta , \gamma )$, and the selected background bands based on keywords extracted from patients’ clinical reports by Natural Language Processing (NLP). We also introduce scalp EEG electrodes reduction by using the electrodes keywords (pre-frontal, frontal, temporal, parietal, occipital, central) extracted from individual’s clinical reports.

The research questions that we aim to answer using out novel approach can be synthesized as follows:

- RQ1: How do the selection of frequency bands $(\alpha , \beta , \theta , \delta , \gamma )$ influence seizure classification?
- RQ2: How do the selection of EEG electrodes (Fp, F, T, P, O, C) influence seizure classification?
- RQ3: How to integrate prior knowledge from experts to build individualized seizure classification models?

In this work, to answer the above stated questions, we (i) present the evidence of background EEG in brain functionality during seizures, (ii) illustrate that selective background frequency bands and EEG electrodes coupling can lead to better seizure classification results, and finally (iii) build a resource-efficient model targeting for individualized seizure classification purpose.

In Sect. 2, we provide background knowledge of the paper. In Sect. 3, we discuss related works. In Sect. 4, we introduce the publicly available dataset used in this work. In Sect. 5, we introduce the design thinking process, the methods including pre-processing using Short-Time Fourier Transform (STFT) and Natural Language Processing (NLP), and the machine learning algorithms. Sect. 6 reports the experiment results, discussed in . Finally, in Sect. 7, we draw conclusions and propose future works.

## Background

In this section, we discuss the medical background of the research and different types of seizures. Specifically, we introduce the “*10-20 system*” standardized EEG electrodes placement, the normal and abnormal seizures, and the classification of different types of seizures.

### EEG electrode reference placement

**Fig. 1:**
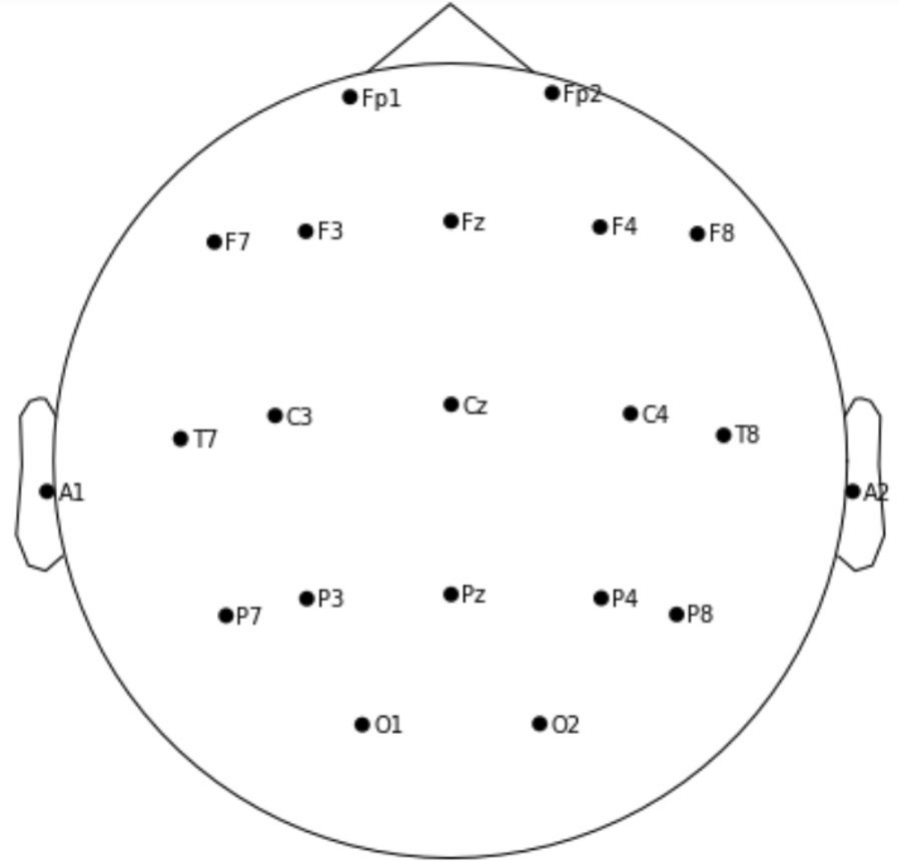
The *10-20 system* for EEG electrodes positioning

Electrodes connect the EEG machine to patients for the recording of electric inputs generated from brain activities. The standardized electrode placement is represented in Fig. 1. It follows the international “*10-20 system*” which has been originally proposed in 1958 [17]. The name of the electrodes consists of two symbols. The first symbol is an abbreviation letter precisely pointing to the underlying six brain zones. The letters include F (frontal), Fp (pre-frontal), P (parietal), C (central sulcus), T (temporal), and O (occipital). Additional electrodes are placed behind the outer ear to record the prominent bone process using the letter A. The second symbol is a number (when on mid-line it is a letter z) specifying the left or right brain cortex: electrodes located on the scalp’s right side are assigned even numbers, and odd numbers are used for electrodes on the left side. Smaller numbers denote positions closer to the mid-line, and larger numbers are farther away spots. Note that electrodes P7 and P8 are placed over the posterior part of the temporal, not the parietal region, and also F7 and F8 electrodes are not only close to the frontal cortex but also the pole of the temporal lobe.

The commonly used reference schemes of EEG electrodes are categorized into two classes, namely unipolar and non-unipolar references [11]. Unipolar references construct a neutral record, including Average Reference (AR), Linked Ears reference (LE), and Reference Electrode Standardization Technique (REST).

AR assumes that neuroelectricity transmits isotropically on a perfect layered spherical head, thus using the average of a finite number of electrodes as a reference. LE reference is based on the assumption that due to the sites lack of electrical activity, the average of the potentials recorded is close to zero between two ears. REST is based on the fact that the same brain sources generate all EEG activities. Non-unipolar references are the potential differences of electrodes, including the bipolar and the Laplacian reference. Bipolar Reference shows the 1st derivative of potentials, which is the difference between two nearby electrodes’ potential. Laplacian Reference show the second derivative of potentials, which is the difference between each electrode’s potential and its nearest four neighbors’ averaged potential.

The advantage of unipolar references is that the changes can be observed directly since it is the potential of the electrodes. The main disadvantage is that they are sensitive to common noise and artifact activity. If one electrode is contaminated, interpretation of activity in the brain area can be difficult. Non-unipolar references are not affected by noise as it is the difference of potentials, but this may attenuate the abnormalities observed in the recordings. If the derivation is zero, e.g., caused by equal effects of cerebral activity around electrodes, the interpretation can be challenging.

### Normal and abnormal EEG

**Fig. 2:**
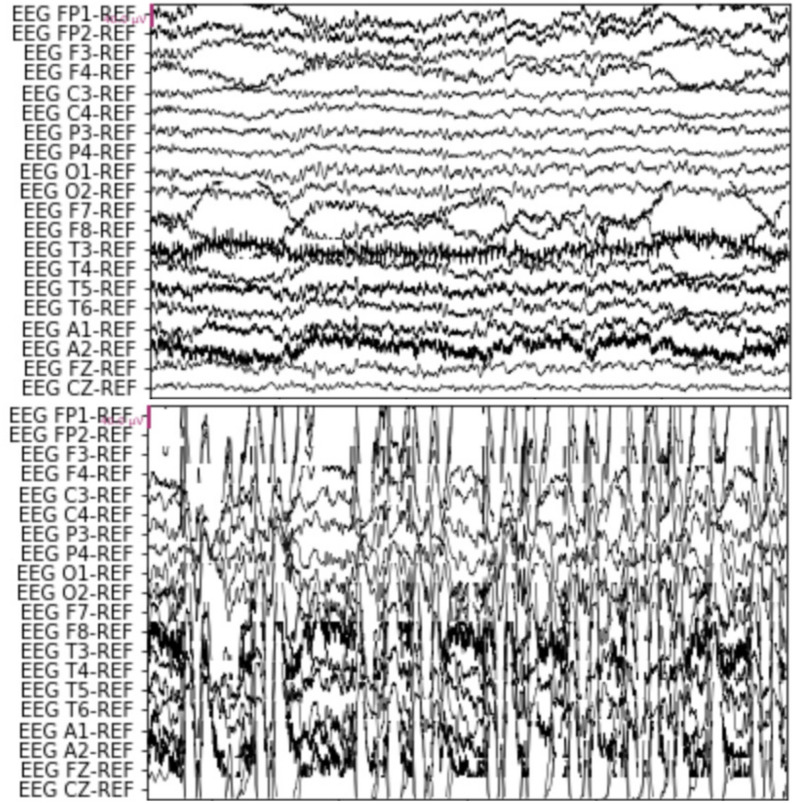
Ten seconds EEG sample of: normal EEG (top), seizure EEG (bottom)

Normal EEGs are measurable both qualitatively and quantitatively. Normal EEG activities appear when people are not affected by any disease. Seizure events consist of abnormal brain activities, formally known as inter-ictal epileptiform discharges (IED). The EEG of IED is characterized by the unusual waveforms that deviate from the normal EEG on frequency, amplitude, morphology, localization, and reactivity. Figure 2 shows 10 s of normal and seizure EEG.

**Fig. 3:**
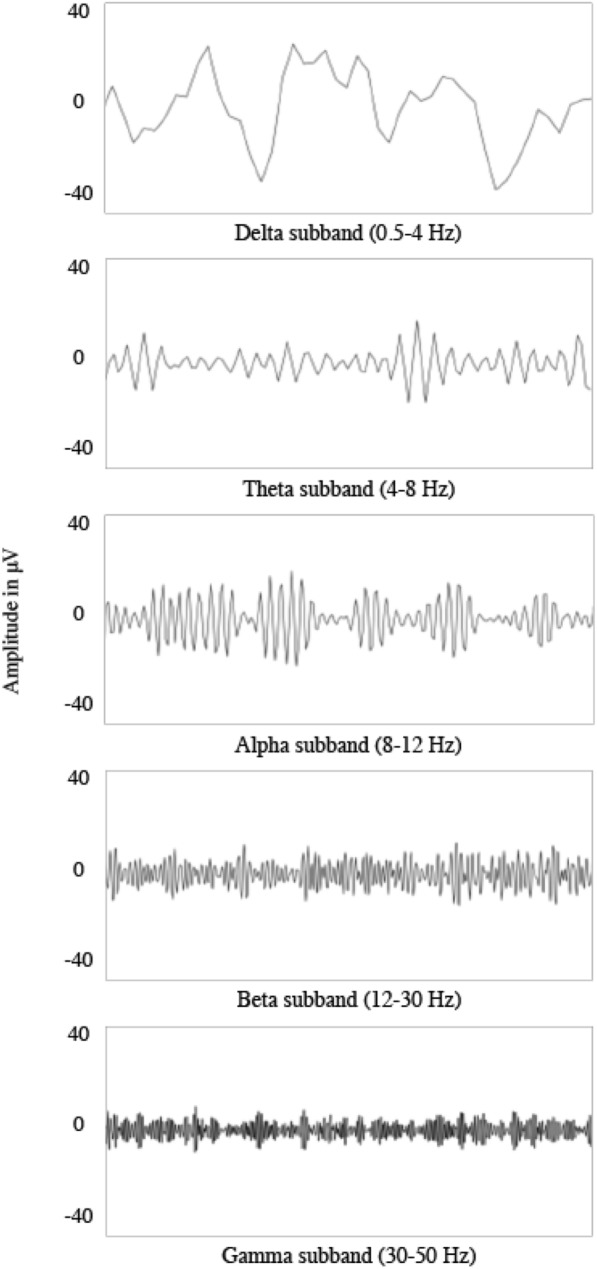
Normal EEG signals with delta, theta, alpha, beta, gamma $(\delta , \theta , \alpha , \beta , \gamma )$ sub-bands

In Fig. 3, the five most common normal EEG activity frequency bands $\alpha , \beta , \theta , \delta , \gamma$ are represented. Each band may have a different interpretation, that can be described as follows:

(i) Alpha rhythm $(\alpha )$: frequency between 8 and 12 Hz. It is more prominent in the occipital regions of an adult brain and can be observed in amplitude during relaxed and eyes-closed wakefulness. When eye-open and mental alert, alpha activities decrease in amplitude and demonstrate reactivity. Alpha variants are the mixture of the alpha rhythm with other rhythms, which have distinct morphology but, in another way, exhibit the same reactivity and localization.
(ii) Beta rhythm $(\beta )$: frequency between 12 and 30 Hz. It is primarily seen in the frontal and central areas of the adult brain. It also exhibits a gradual increase with age in the frequency for children. Beta activity is triggered by alertness and vigilance, suppressed by voluntary movements.
(iii) Theta rhythm $(\theta )$: frequency between 4 and 8 Hz. It is prominently seen in the central, parietal, and temporal parts of the left side scalp recording. Theta rhythm can reflect the abnormal activity in adults during wakefulness and is frequently observed in adults in sleep state.
(iv) Delta rhythm $(\delta )$: frequency between 0.5 and 4 Hz. It is most predominantly found in adults frontally and in children posteriorly. Delta waves are associated with the deepest levels of the sleep stage and have a healing effect on the body and brain.
(v) Gamma rhythm $(\gamma )$: frequency between 30 and 50 Hz. It is seen in the cerebral cortex with cognitive and motor activities. Visual stimulation and meditation could increase the amplitude of gamma rhythms. It is often observed in the seizures ictal phase and prevalent in seizure onset. Altered gamma oscillations are regularly detected in brain disorders like Alzheimer’s disease besides epilepsy.

Abnormal EEG activity is often prevalent in people with neurological or other diseases and absent from normal individuals. IED is the abnormal synchronous electrical discharge that originates in epileptic focus with a group of misfunctioning neurons [18]. Sharps and spikes are the prominent abnormal EEG waveforms and manifest as pointed peaks, serving as biological markers for either focal or generalized epileptogenesis. Spike waves are transients often exhibit between 20 and 70 ms. Sharp waves are similar but last longer with typical duration of 70–200 ms. Besides duration, sharps and spikes can have varying waveforms, like the voltage, frequencies, etc. Their occurrence can be single or repetitive, and distribution can be focal or general. The appearance of sharps and spikes is asymmetric, with initial deflection primarily as a sharper slope. The observation can be isolated waveforms or can be followed by slow waves. The subtypes can be divided by multiple ways. For example, by localization, there are temporal/centrotemporal/occipital/generalized spikes and sharp frontal waves; frequency spikes and sharps are associated with various frequency ranges, like 6-Hz spike-and-wave, polyspikes, and 14- and 6-Hz positive bursts, etc. The spike-and-slow-wave complex is the occurrence of a spike followed by a longer duration slow-wave, with varying frequency and amplitude and often distinct from the underlying background. A sharp wave can be the initial waveform rather than a spike. A sharp- and slow-wave complex is identical to the spike- and slow-wave complex, except that a sharp-wave succeeds the slower and broader wave. In these discharges, the slow-wave that follows may symbolize inhibition and subsequent hyperpolarization of cortical neurons, which accompany the initial synchronous depolarization [19]. For epilepsy patients, the above abnormalities are routinely observed between seizure periods and suggest an underlying propensity toward seizures; nevertheless, the abnormalities during a seizure do not result in observable clinical behavior for certain.

### Seizures types

Seizures and epilepsy are classified from International League Against Epilepsy (ILAE) using modern era’s terminology and concepts [20]. The two broader types are defined as generalized and focal seizures. Generalized seizures arise in neuronal networks distributed bilaterally, while focal seizures are limited to one hemisphere. Seizures may propagate from partial to generalized state, when the neuronal network is initially partly altered and may became complete dysfunctional at a later stage. Table 1 reports a selection of seizure categories, together with their symptoms descriptions. For clarity of presentation, the list is partial as it includes only the seizure types included in the dataset used in this work.

**Table 1:** Seizure type description

Seizure types	Seizure subtypes	Description
Generalized seizure (GNSZ)	Absence seizure (ABSZ)	Short and brief disturbance of consciousness
	Myoclonic seizure (MYSZ)	Brief and sudden arrhythmical jerks of muscles
	Atonic seizure (ATSZ)	Sudden loss of muscle tone in limb, neck, and trunk
	Tonic seizure (TNSZ)	Sudden stiffness of the limbs or trunk
	Clonic seizure (CNSZ)	Rhythmical jerking/shivering of muscles
	Tonic–clonic seizure (TCSZ)	Bilateral symmetric convulsive movements of limbs with the impediment of consciousness
Focal seizure (FNSZ)	Simple partial seizure (SPSZ)	Awareness is retained and patients are able to describe motor/sensory
	Complex partial seizure (CPSZ)	Consciousness is impaired and patients are not able to respond to tactile/verbal stimuli

### Seizure classification with EEG

The abnormal EEG recordings of patients who suffer from seizures contain four states: pre-ictal, ictal, inter-ictal, and post-ictal. Pre-ictal and post-ictal are the signal portions before the seizure occurs and after the seizure diminishes, respectively. Inter-ictal is the abnormal signal activities between epileptic seizures, and ictal is the abnormal signals during an epileptic seizure [21].

The electrographic signature of a seizure is composed of spikes and sharps complexes and other abnormal activities that can be inspected over a longer duration compared to its exhibits during inter-ictal periods. Occasional transient waveforms are the signature of inter-ictal activities in EEG. It can exhibit either isolated spikes, sharps, or spike-wave complexes. IED generally supports the diagnosis of seizure disorders such as:

(i) Partial seizures: EEG in partial seizures have two or more distinct phases, which are metamorphic. The common pattern consists of a series of spike- and sharp-waves, mixed with rhythmic waves, also with amplitude attenuation. The frequency and amplitude change dynamically of the waveforms when the seizure spreads in brain regions. At the ends of seizures, the frequency of sequential spikes or rhythmic waves will diminish to a slow spike-wave pattern. Temporal lobe seizure often with initial alpha or theta frequency range with a lesser proportion of slower wave occurring. Extra-temporal seizures frequently start with the beta band. The metamorphic patterns often follow post-ictal slowing delta, suppressing or activating focal spikes. Moreover, the electrodecremental events observed focally can localize the seizure onset zone. It also reflects high-frequency firing or intense neuronal depolarization. However, generalized electrodecremental events that are not ictal prior to focal seizures may epitomize cerebral alter that lead to focal seizures. Note that simple partial seizures with sensory symptoms rather than motor symptoms may not be distinguished in the EEG activities up to 80% of the time. But using more close-spaced electrodes, the ictal can be recognized.
(ii) Generalized seizures: absence seizures have isomorphic and stereotyped features. The frequency and amplitude will change with seizure progression, though. For example, spike-wave discharges may start with 3.5 to 4 Hz at onset and diminish to 2 to 3 Hz, and the spike amplitude will also reduce at the subsequent of seizures. Diffuse polyspike wave complexes can precede tonic–clonic seizures. Ictal signals during the first phase often have generalized attenuation of rhythmic waves and increase in voltage gradually, then evolve into polyspikes. The second phase often shows slow waves mixed with paroxysmal spike activities. Then there is the gradual recovery of rhythms following generalized attenuation in the post-ictal period. Tonic seizures exhibit generalized paroxysmal fast activities or diffuse voltage attenuation with associated sharp and slow-wave complexes. Myoclonic seizures often companies with 10 to 15 Hz polyspikes and slow waves. Generalized atonic seizures may show 2–3 Hz spike-wave discharges or may not be associated with any EEG change.

However, the EEG may not capture all of the ictal activities because the technique limitations. For example, the skull and scalp may filter out some frequency waveforms, and the placement of recording electrodes may shift the distance and orientation of the seizure focus. Despite the limitations, seizures recorded by EEG can provide helpful information regarding the seizure type and focus.

## Related work

The advance in machine learning boost its application in biomedical related field. Seizure detection have been widely studied from different perspectives and using different features. For seizure detection the discriminative features consist of morphological, biological and rhythmical features. Morphology features are used to detect and differentiate the components of amplitude and fundamental frequency in EEG waveform [4, 22]. Biological features, such as synchronization likelihood, help to distinguish epileptic seizure activities from non-epileptic background activities [23]. Rhythmical characteristics include features in time, frequency or combined domain. In addition, to fit the non-linear and non-stationary nature of brain signals and capture the changes reflected in EEG, research has been made to include magnitude components of signal in time domain [24], spectral representation, magnitude from signals in frequency domain [5, 25], and time–frequency domain features [26, 27].

In this section, we first introduce previous works of frequency bands’ role related to brain functionality for abnormal EEG detection. Later we will present the seizure classification approaches for EEG channel selection with noise and computational power reduction.

### Frequency bands selection

Traditional signal processing techniques are being applied for extracting the morphology patterns that constitute an epileptic seizure. Epileptic EEG recordings of the spike and sharp-wave complexes can be easily distinguished by morphological characteristics of waveforms’ amplitude, shape, and duration. The grounding idea is the geometric difference between spikes and background activities such as the slope’s distinctive attributes and sharpness, height, and length of waves. In morphological analysis, EEG waves are often decomposed to smaller physical parts like two opposite half-waves [28–30], and structure divergence of background activities and spikes complex can be observed.

One of the first automated seizure detection algorithms developed by Gotman in 1982 [4] analyzed signals morphologically. Their system first breaks down EEG signals to half-waves and searches for morphology-based features, particularly the epileptiform spike- and sharp-waves in the recording of 16 bipolar channels. They applied frequency thresholds between 3 and 20 Hz and relative amplitude with a dynamic baseline of background window in time domain features. A seizure is declared when the degree of rhythmicity for at least two channels exceeds the thresholds and lasts for four seconds. The algorithm successfully detected seizures with rhythmic activities with a determined threshold, but the algorithm fails when seizures consist of a mixture of frequencies or amplitude. Moreover, since rhythmic activity can be induced by normal or artifact bursts other than pathologic, the algorithm’s detection may not be associated with seizures. Further studies found that the key to morphological analysis is to select a proper filter, restraining the background activities while retaining spikes. Nishida et al. [22] presented a detection method using a morphological filter, with the basic algorithm of open-closing morphological operation and structure elements of second-order polynomial functions. Pon et al. [31] proposed a mathematical morphology approach plus wavelet transform to detect bi-directional spikes with a circle structure element. Xu et al. [32] improved morphological filter with differences of their geometric characteristics to separate spikes from background activities. EEG enhancement strategies have been introduced in various works with the aim to better detect the spikes, to increase the candidate spikes, to minimize the missing seizure events, and to minimize the false selection [33–36].

The analysis of abnormal EEG spikes and sharps is the gold-standard to diagnose seizures, while the background activity has been scarcely studied. The main reason is that by applying visual inspection of brain signals the abnormalities in background EEG $(\alpha , \beta , \theta , \delta )$ cannot be distinguished. However, studies have shown that the background activities contain vital information about function and dysfunction of the brain in human epilepsy [7]. The seemingly normal EEG background bands may include evident abnormalities that can be used as a significant prognostic tool.

Alpha rhythm slowing in epilepsy was observed to be associated with mental deterioration [37]. Peak alpha frequency variability has been found between epilepsy patients and the control group, with a lower alpha frequency in the epilepsy group [38]. When the dependencies on antiepileptic drugs are ruled out, the epilepsy biomarker was sensitive to alpha rhythm abnormalities [39]. Alpha spectral power shifts from high to low in both focal and idiopathic generalized epilepsy patients compared to healthy subjects, indicating poorer seizure control [40]. Alpha oscillations have been used as an index to the cortical–subcortical brain network function abnormally in photosensitivity epilepsy patients [41].

Theta signals association with epilepsy has been demonstrated as well. Theta bands have been found to be positively related to the number of epileptic seizures in patients with brain tumors [42]. When monitoring inter-ictal activities and theta oscillations in parallel, spatial deficits correlated with a decrease of theta power while non-significantly related to inter-ictal activities in rats model of temporal lobe epilepsy [43].

The delta oscillations have been proved to have a high correlation with epilepsy. The asymmetry of the delta signals can be used as a biomarker to identify the epileptogenic zone [44]. Temporal intermittent rhythmic delta oscillations can be a signature of focal epilepsy [45, 46]. The delta slow waves are often prevalent in patients with uncontrolled seizures [47], and inter-ictal regional delta slowing has also been found to correlate positively with temporal lobe epilepsy patients’ surgical outcomes [48, 49].

### EEG montages selection

The EEG montages refer to the electrodes located on the patients’ scalp. Montages are named consistently with the locations of brain cerebrum, with abbreviations Fp (pre-frontal), F (frontal), C (central), P (parietal), T (temporal), and O (occipital). A large number of montages (often ranging from 19 to over 100) are used when performing different tasks such as emotional response analysis, sleep recordings and drug effect diagnosis. For efficient analysis, it is vital to pick out the montages which carry the most discriminative information. Neurologist’s selection with prior knowledge demonstrates the effectiveness of seizure detection with only a small number of montages [12, 13]. In computer science, the selection of EEG montages is widely studied [10] for faster seizure detection and noise removal. However, the underlying principles guiding the montage’s selection by neurologist and computer scientist is different. Computer scientists generate a subset of electrodes from whole set using various statistical measures like variance and entropy as well as using techniques such as power spectral estimation and wavelet transform. The selection made by neurologist is directed by the experience in diagnosing the particular disease as well as by the underlying knowledge of which brain areas generated the abnormalities in electric signals.

The montage selection has manifold objectives including the reduction of model computational complexity and model overfitting. By utilizing the montage that contains significant features, the associated brain areas of the montages can be identified. The specific regions of the brain contain vital information about the Seizure Onset Zone (SOZ) that may reflect where IEDs originate.

Several studies have shown the functional and topological change of the brain network during the inter-ictal, and ictal phase [50–52]. Burns et al. [53] studied the network structure of epilepsy patients’ brains by constructing a graph with intracranial electrocorticographic (ECoG) recordings. The nodes are electrodes, and edges are node pairs with associated frequency bands’ coherence weight. They found that the brain network dynamics can be characterized into a finite set of states, where the seizures’ progress can be defined using a consistent sequence of the sub-states. Moreover, during the sub-states, the nodes are separated where a subset of nodes are isolated, and this subset of nodes can identify SOZ with high sensitivity and specificity. Martinet et al. [54] analyzed the brain dynamics during seizures at microscopic and macroscopic level. Their results indicate that the distance is a vital factor: the electrical voltage of activities decrease with longer distances in spatial scales coupling, and the coherence of waves propagation increase is dependent on distance.

With the supporting evidence of seizure related to a specific part of the brain cortex, further studies on seizure identification unitized the information of brain areas with recording montage references. Using a subset of electrodes to distinguish seizures have been proved from both vivo experiments [12, 13] and signal processing by machines [14–16] to be a plausible approach. With the ultimate purpose of implementing the seizure detection models in wearable or invasive devices, machine learning-based automatic channel selection is targeted to reduce the computational cost of models. The typical procedure in channel selection consists of three steps: electrode subset generation, subset evaluation and result validation. Subset are first generated and then evaluated. Subset generation has been explored using complete search, sequential search, or experts’ generated. In literature five main approaches can be found for subset generation: filtering, wrapping, embedded, hybrid, and human-based techniques [10]. Truong et al. [15] select the channels by comparing the spectral power and correlation in both frequency and time domains between the electrodes pairs. Their method outperforms the other methods without channel selection two times faster and maintaining the same level of accuracy as well as area under curve. Ibrahim et al. [16] used a statistical approach in time-domain signals for channel selection. They sliced the data by using a sliding window to each 10 seconds non-overlap segments, then the probability density functions (PDFs) of derivatives, local means, local variances, and medians is calculated for each segments. The resulting multiple bins PDFs are studied individually and compared with the pre-defined thresholds in prediction and false-alarm probability. After the comparison, the bins are selected from certain channel for seizure prediction.

## Dataset

In the past, seizure prediction studies using EEG signals have been limited due to insufficient standardized and qualified data [55]. The EEG data have often been acquired from Intensive Care Unit (ICU) [56], presurgical inpatient [57], animals [58], and implantable devices [59]. Data usefulness was limited by the relatively short recording duration and inter-ictal time sampling of patients’ and animal models’ concerns. Furthermore, the data were primarily held at the institutions where it was acquired and not made available for the community to use.

Recently, the opening and sharing of longer-lasting and high-quality publicly accessible chronic EEG datasets, such as the CHB-MIT Dataset [60], the UPenn and Mayo Clinic seizure detection dataset [61], and the Temple University Hospital (TUH) Dataset [62], has made possible the advance of seizure prediction algorithms.

The TUH Seizure Corpus (TUSZ) assembles EEG data in clinical settings from archival and ongoing records at Temple University Hospital [63]. TUSZ is the most extensive open-source corpus both in terms of quantity and heterogeneity, with a wide variety of seizure morphology in aspects of frequency, amplitude, and onsets. The TUSZ is organized by patient and session in a hierarchical file tree structure. Each patient folder is composed of sub-folders corresponding to their recording sessions. Each session has EEG signals stored in a standard European Data Format (EDF) and the corresponding clinician reports in text format (TXT) collected by certified neurologists.

### EEG data

TUSZ v1.5.2 released in May 2020 comprises 3050 seizure events. More specifically, the EDF files include the following metadata: anonymized patient ID, age (in years), gender, recording date, and per-channel information (labels, sample frequency, channel physical dimension/min/max, prefiltering channel conditions). It is also worth noting that the EDF files include a varying quantity of channels and sampling rate [62], where channels consist of EEG-specific channels coupled with supplemental channel information such as detected bursts and photic stimuli. EEG signals have been sampled at 250 Hz, 256 Hz, 400 Hz, or 512 Hz.

The EEG data of each patient include information about: (i) assigned numbering: patient ID, the session numbers of each patient, the file numbers of each session, filename (consist of electrode reference, patient, session, and file IDs); (ii) EEG type and subtypes: Epilepsy Monitoring Unit (EMU), Intensive Care Unit (ICU) including eight subtypes (burn unit, cardiac intensive care, intensive care unit, neuro-ICU facility, neural surgical ICU, pediatric intensive care unit, respiratory intensive care unit, surgical intensive care unit), inpatient but not ICU (Inpatient) including three subtypes (emergency room, operating room, Inpatient but not ICU or outpatient), routine EEGs (Outpatient), EEG report is not informative (Unknown); (iii) EEG label: LTM-or-Routine, Normal-or-Abnormal; (iv) description of seizures: number of seizures/file, number of seizures/session, seizure start time, seizure stop time, seizure type.

In this study, we use four selected categories: filename for extracting the data, seizure types for clusters, seizure start and stop time for ictal/inter-ictal label. TUSZ contains three types of electrode reference, all constructed from neutral record listed in Table 2. Here the 03_tcp_ar_a is 01_tcp_ar without electrode A1, A2 that connect to the left ear and right ear. The three types of electrode references used to record brain signals in TUSZ have fundamental hardware and interpretation differences as described in . This variability in referential montages may affect the performance of machine learning algorithms [64].

**Table 2:** Electrode reference description and events count by seizure type in TUSZ

Electrode reference	Seizure types	Seizure events
01_tcp_ar	GNSZ	428
	ABSZ	2
	MYSZ	1
	TNSZ	62
	TCSZ	28
	FNSZ	1070
	SPSZ	52
	CPSZ	138
02_tcp_le	GNSZ	87
	ABSZ	97
	MYSZ	2
	TCSZ	16
	FNSZ	231
	CPSZ	83
03_tcp_ar_a	GNSZ	68
	TCSZ	4
	FNSZ	535
	CPSZ	146

Statistical analysis has been performed to investigate the latent divergence caused by three electrodes reference in TUSZ. For selecting the suitable subjects that can exclude the variation caused by individual difference, seizure type, and health state, filters are applied as follows: (1) subjects need to have recordings from three different electrode references for parallel comparison; (2) subjects need to have same EEG type, which means same health state; (3) recording files do not contain seizures, as seizures alter the recorded signals. Two particular subjects with patient ID 4671 and 6514, both with EEG type as ICU, have been selected for this analysis.

The statistical variation is measured in three domains of amplitude, time correlation, and frequency correlation with mean, standard deviation (STD), minimum, and maximum. Before the measurements are taken, data are pre-processed by resampling and unit scale normalization. Fast Fourier transform (FFT) is applied to transform data in the time domain to the time–frequency domain. The correlation in time and frequency is calculated by taking eigenvalues on the correlation coefficients matrix. The result is provided in Table 3. From the table, it is clear that for the three types of electrode references, the mean and standard deviation greatly deviated in the amplitude domain, but the basic statistics remain at the same level in time and frequency correlation. The results suggest that the recordings from the three types of electrode references have high inter-variability and low intra-variability. Thus, the classification is performed under each electrode reference, respectively.

**Table 3:** Basic statistics of amplitude, time correlation and frequency correlation

	Amplitude				Time correlation				Frequency correlation			
	Mean	STD	Min	Max	Mean	STD	Min	Max	Mean	STD	Min	Max
01_tcp_ar	− 7.4	219.4	− 5482	5482	− 0.04	0.29	− 0.71	0.68	− 0.05	0.05	− 0.11	0.17
02_tcp_le	− 35.2	55.42	− 981	4100	− 0.04	0.22	− 0.53	0.65	− 0.04	0.18	− 0.32	0.77
03_tcp_ar_a	1.31	173.6	− 5482	5482	− 0.04	0.25	− 0.71	0.72	− 0.05	0.11	− 0.21	0.54

### Textual data

**Fig. 4:**
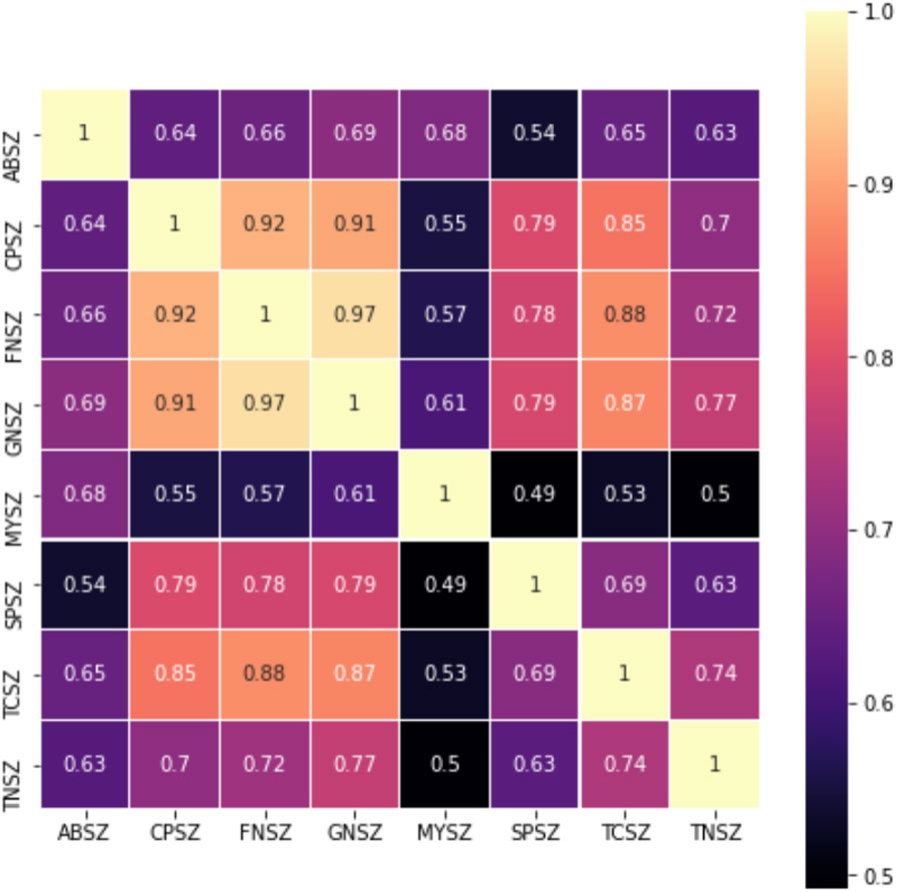
Similarity matrix heatmap of seizure types by clinical reports

The clinical reports provided together with the signal file in TUSZ are critical for seizure diagnosis. This feature renders the TUSZ unique of its kind as no other dataset provides the information. The clinical reports document medics’ knowledge of the patients. The information given contains introduction, clinical history, medications, record description, and seizure impression. Primary exploration and further feature extraction have been applied to textual data to utilize the text information delivered in the reports. Term Frequency–Inverse Document Frequency (TF-IDF) in Natural Language Processing (NLP) has been carried out to calculate pairwise similarity among the clinical reports of different seizure types, the calculated similarity heatmap is shown Fig. 4.

Moreover, top frequency words by TF-IDF are recorded and compared for every seizure type. Full list of top 50 keywords can be found in Tables 11 and 12 in Appendix. From the list four common observations can be made based on the terms with high occurrence: (1) general words: seizure, activity, EEG, clinical, record/recording, etc.; (2) descriptive words of seizures: spike, sharp, complex, discharge, etc.; (3) location words: right, left, frontal, temporal, hemisphere, posterior, anterior, etc.; and (4) background signal frequency words: alpha, beta, theta, delta $(\delta , \theta , \alpha , \beta )$.

For observations (1) and (2), it is self-evident these words are frequent in clinical reports to describe the abnormal brain signals which lead to a seizure event and no more information can be derived. Nevertheless, the (3) and (4) are the informative terms that carry the knowledge of what the medics are inspecting on the given EEG signals and how they would ultimately diagnose the seizure. For example, some of the descriptions of the record from the clinical reports are quoted here: “A prominent increase in beta noted at 3 a.m.”, “A status epilepticus pattern with prominent epileptiform activity from the right occipital and temporal region.”, “There is a posterior dominant rhythm of 8 Hz, 30 to 50 V with a small amount of low voltage, frontocentral beta activity.”. The description reflects the thinking process of experts when they examine the EEG data. To mimic the thinking flow of medics when dealing with seizures, we designed a classification framework that aims to classify the ictal/inter-ictal state in a similar way. The process is introduced in Sect. 5.

## Methodology

This study aims to mimic the reasoning process of medics when they diagnose seizures and integrate the expert’s knowledge into the classification process when classifying ictal/inter-ictal signals. Ultimately, the goal is to build a personalized seizure classification system. Not all the EEG signals are essential when identifying seizures from observation and using common sense. In many studies, the focus is on identifying signs of seizures from the seemingly abnormal waveforms like the spike and sharp waves. Nevertheless, as discussed in Section , previous works have demonstrated how the seemingly normal background bands play an important role when diagnosing seizures and how different areas of the brain are activated when a seizure happens.

**Fig. 5:**
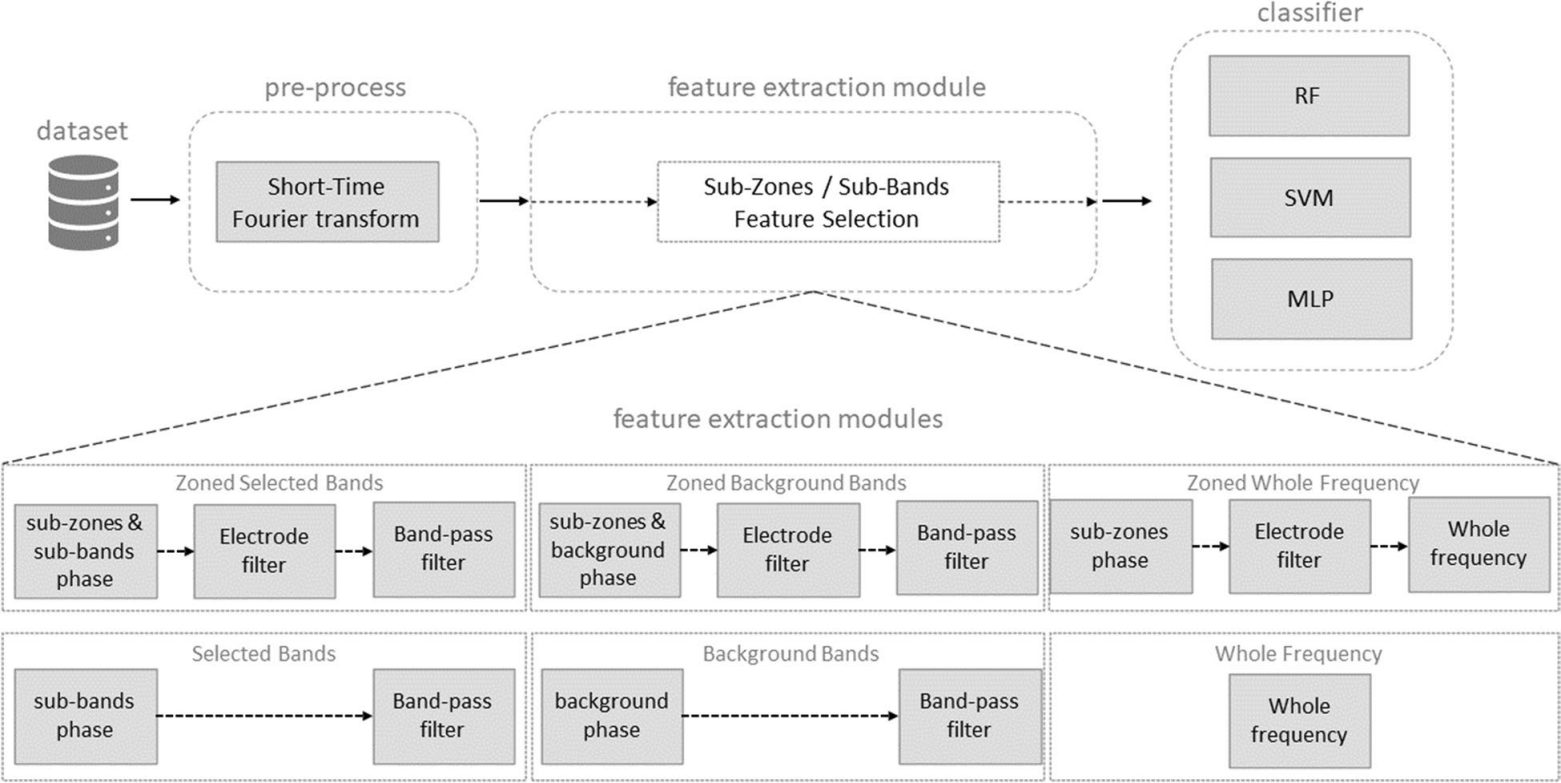
Classification process flowchart. Feature extraction can be one of six different modules and may include different levels of sub-zone and/or sub-band filters

Experiments are designed with pre-process of input data, including time–frequency data transformation, language-signal mapping, inputs selection, and classifier evaluation as shown in Fig. 5.

### Time–frequency transformation

Several methods are available to perform time–frequency transformation of signals, such as wavelet transform and short-time Fourier transform (STFT). Previous studies have shown that STFT should be preferred over wavelet transform for determining epileptic seizure activity in real-time [65]. STFT is used for spectral analysis of EEG signals and it can transform signals between the time and frequency domain due to its timeshift invariant. Specifically, for every chunk of signals in the time domain by sliding window, the one-dimensional Fourier transform is applied, resulting in a two-dimensional time–frequency representation of the signal. Sliding windows are used to partition time-series data into a finite number of segments. The window length is the trade-off between spectral and temporal resolution, where the longer window preserves more spectral information and less temporal information. The window length is analyzed with sliding action in time and the changes of spectral behaviors in each block are observed locally. Different sliding window lengths are tested and determined to balance time and frequency resolution in this study. Finally, fixed-width sliding windows of 2 s with 50% overlap are implemented in this study. The number of samples per window is related to the specific sampling rate (250 Hz, 256 Hz, 400 Hz, or 512 Hz) of the specific recording file.

### Natural language mapping of signals

Frequency domain EEG signals are further filtered to detect seizures based on knowledge and build a resource-utilized classifier. More specifically, information from the clinical reports is extracted and mapped on the EEG signals.

Basic Natural Language Processing (NLP) techniques are used. First, the noise like punctuation is removed, then sentences are tokenized into a list of words. After that, each token was converted to lower case for more accurate selection purposes. Finally, two lists of keywords are targeted: background frequency bands (alpha, beta, theta, delta, gamma) and zones of brain areas (pre-frontal, frontal, temporal, parietal, occipital, central). At the end of the NLP pipeline we will produce the two lists of words from each patient documents if the above keywords and their variants are present.

The signals are then mapped using the extracted lists of sub-bands and sub-zones for the classification process. The band list mapping is guided by the frequency range of alpha (8–12 Hz), beta (12–30 Hz), theta (5–8 Hz), delta (0.1–5 Hz), and gamma (30–50 Hz). The zone list is guided by the annotation of the *“10-20 system”* of Fp (pre-frontal), F (frontal), C (central), P (parietal), T (temporal), and O (occipital). The mapping is performed at the individual patients’ level, and the corresponding frequency signals can be extracted from individual patients’ EDF files. The extracted keywords cluster of sub-bands and sub-zones by each seizure type are shown in Sect. 6.1.

### Inputs selection

In order to compare the efficiency and resource utilization of the mapped signals, six types of input data were tested:

1. Zoned selected bands: selected electrodes and selected frequency bands extracted from clinical reports;
2. Selected bands: input selected frequency bands extracted from clinical reports;
3. Zoned background bands: selected electrodes and background frequency bands;
4. Background bands: input background frequency bands;
5. Zoned whole frequency: selected electrodes and whole frequency range without any selection.
6. Whole frequency: input whole frequency range without any selection.

The “Zoned” feature extraction (approaches n. 1 and n. 3) use selected electrodes (Fp, F, C, P, T, O) corresponding to the brain zones (pre-frontal, frontal, central, parietal, temporal, occipital). The “Selected Bands” feature an extraction (approaches n. 1 and n. 2) use selected frequency sub-bands (selection of $\delta , \theta , \alpha , \beta , \gamma$). The approaches n. 3 and n. 4 evaluate the role of all background band signals $(\delta , \theta , \alpha , \beta , \gamma )$ in seizure and normal conditions. Approach n. 1 evaluates the classification power using the data points as limited as possible. Approaches n. 6 use all available data points without further selection, including abnormal seizure waveforms like spikes and sharps.

### Classification models

In this work, we apply both traditional machine learning and deep learning models for the seizure classification. The traditional machine learning models Random Forest (RF) and Support Vector Machines (SVM) are utilized due to their ability of better handle imbalanced datasets, the need of fewer resources. The deep learning model include a multi-layer perceptron (MLP) artificial neural network with various topologies.

*Random Forest (RF)* [66]: The RF classifier is formed by a combination of tree classifiers. Each of the trees is formed by a random vector selected separately from the input vectors. Each tree appoints a unit vote for one most sampled classes to classify an input vector. Because RF is a tree-based ensemble where the final classification is then made of a majority vote yielded by each ensemble of trees, a RF may be defined as a group of Decision Tree (DT). Each instance in the dataset is classified by each tree. The final classification decision is made by averaging the probabilities of class assignment by produced trees. An unlabeled instance is evaluated by all DTs created in the ensemble, and each DT votes for a class, the most voted class will be the final classification decision of the instance. The tree’s growth each time towards the maximum depth using a combination of data features. Thus, by growing RF to the set number of trees, the algorithm generates trees with high variance and low bias.

*Support Vector Machine (SVM)* [67]: The SVM decides the separation between two classes by separating observations with an optimal hyperplane using statistical theory. In linear separable classes, the optimal hyperplane is the one that gives the widest margin of the two types. Margin is measured by the vectors that are closest to the hyperplane. Therefore, the vectors are named ‘support vectors’ and are have a shorter distance from the hyperplane than other vectors in the class. SVM focus on maximizing the margin and minimizing the misclassified vectors. A self-defined tuning parameter in practice restrains the trade-off between them. Maximizing the margin can be solved using optimization techniques like standard Quadratic Programming (QP). In non-linear separable classes, the optimal hyperplane is determined in a higher-dimensional feature space where the classes can be separable linearly. Kernels project functions that allow SVM to find optimal hyperplanes in higher-dimensional space without knowing the explicit transformation and construction. A Radial-Basis Kernel (RBF) is one of the most popular non-linear mapping functions. The decision region of an RBF can be the union of several disjoint areas. Since the determination of the optimal hyperplane in its associated high-dimensional feature space yields non-linear decision boundaries that may be necessarily discontinuous.

*Multi-Layer Perceptron (MLP)* [68]: The MLP is an artificial neural network feed-forward model consisting of three types of layers: one input layer, one output layer, and at least one hidden layer. Each layer is composed of simple computational units called neurons. Neurons between layers are interconnected. The input layer receives the data and passes the data to the first hidden layer. The output layer could be a list of categories or signals mapped to the input data. The hidden layers minimize the loss of the model by adjusting each neuron’s weights and biases and extracting the salient features that have a predictive power of the output. Nonlinear and continuously differentiable activation functions are applied at each hidden layer to transform the data and decide whether the neuron needs to be activated or not. Rectifier Linear Unit (ReLU) is now used as a default activation function. It has a lower running time and reduced likelihood of vanishing gradient, which is a problem often seen in other activation functions like sigmoid and hyperbolic tangent (tanh).

## Experiments

In this section we present the experiment results. The results include the aggregation of keywords extracted with NLP, the seizure classification with six different inputs, and the ablation study.

### Natural language keywords aggregation

Table 4 reports on the sub-band keyword’s aggregation for each seizure type. Table 5 reports the electrode reference brain zones aggregation of keywords. We count the binary appearance of keyword in each clinical report (e.g., if a keyword occurs more than once in one report the count is one). Each seizure type contains a varying number of clinical reports that affects the number of discovered keywords. A useful additional indicator for analysis is the proportion of the keywords of specific sub-band and electrode reference zones indicated by the relative percentage in brackets.

From the natural language processing we obtain interesting information about the clinical reports. We can find that the GNSZ clinical reports are characterized by Theta and Beta frequency bands together with Temporal (T) and Frontal (F) electrode references. FNSZ clinical reports point to Delta and Theta bands maintaining the same Temporal (T) and Frontal (F) brain reference zones with varying relative percentages comparing to GNSZ. We can also notice that the Gamma frequency band is not present in many reports, just a small percentage (0.9%) of the big set FNSZ contains this information. The brain zone references electrodes are evenly present in different reports with the exception of pre-frontal (Fp) electrodes that are only found in 0.2% of FNSZ reports.

In the following experiments, a fixed number of electrodes for every patient have been selected as grounding. Electrode references 01_tcp_ar and 02_tcp_le contain 21 electrodes in total and five electrodes (A1, A2, CZ, C3, C4) have been used as fundamental for all the patients. The electrode reference 03_tcp_ar_a contains 19 electrodes in total and three electrodes (CZ, C3, C4) have been selected.

**Table 4:** Clusters of frequency bands selected from keywords in clinical reports

Seizure type	Number of frequency bands (relative %)				
	Alpha	Beta	Theta	Delta	Gamma
GNSZ	35 (14.6)	69 (28.75)	50 (35.8)	86 (20.8)	0
ABSZ	5 (20.0)	5 (20.0)	6 (24.0)	9 (36.0)	0
MYSZ	3 (50)	0	1 (16.7)	2 (33.3)	0
TNSZ	0	28 (73.7)	1 (2.6)	9 (23.7)	0
TCSZ	9 (20.9)	15 (34.9)	11 (25.6)	8 (18.6)	0
FNSZ	254 (15.6)	299 (18.3)	524 (32.1)	538 (33.0)	15 (0.9)
SPSZ	0	5 (33.3)	5 (33.3)	5 (33.3)	0
CPSZ	55 (28.9)	21 (11.1)	73 (38.4)	41 (21.6)	0

**Table 5:** Clusters of electrode references selected from keywords clinical reports

Seizure type	Number of brain zone reference (relative %)					
	Fp	F	T	P	O	C
GNSZ	0	48 (27.1)	76 (42.9)	7 (4.0)	21 (11.9)	25 (14.1)
ABSZ	0	31 (47.0)	7 (10.6)	6 (9.1)	10 (15.2)	12 (18.2)
MYSZ	0	2 (100)	0	0	0	0
TNSZ	0	20 (24.4)	27 (32.9)	8 (9.8)	0	27 (32.9)
TCSZ	0	22 (35.5)	19 (30.6)	1 (1.6)	6 (9.7)	14 (22.6)
FNSZ	2 (0.2)	348 (25.6)	397 (29.2)	156 (11.5)	218 (16.1)	237 (17.5)
SPSZ	0	0	1 (14.3)	1 (14.3)	4 (57.1)	1 (14.3)
CPSZ	0	40 (24.1)	48 (28.9)	21 (12.7)	15 (9.0)	42 (25.3)

### Seizure classification

#### Experimental settings

In the classification experiments presented in this work, the *“positive class”* is the inter-ictal EEG phase where no seizure happens, and the *“negative class”* is the ictal EEG signals where seizures are observed. Training of the classifiers has been performed using fivefold cross-validation, where the dataset is split in 5 non-overlapping parts. For each fold four parts are used for training of the classifier and one for validation of the results. For additional robustness of the results, the evaluation metrics are measured with the average of each fold.

#### Evaluation metrics

Seizure detection is a binary classification problem. The experiments’ results have been evaluated using accuracy (ACC), area under curve (AUC), sensitivity (TPR), and specificity (TNR) scores. Given the basic statistics of true positive (TP) that measures when the predicted seizure corresponds to a seizure in the dataset, false positive (FP) measurement of the predicted seizure is non-seizure in the dataset, true negative (TN) measuring the predicted non-seizure that is non-seizure, and false negative (FN) that measures when the predicted non-seizure is a seizure. The evaluation metrics can be calculated using above measures as follows:

- Accuracy (ACC): $\frac{TP + TN}{TP +TN + FP + FN}$, the ratio of predicted seizures and non-seizure to the total number of samples;
- Sensitivity/true positive rate (TPR): $\frac{TP}{TP + FN}$, the ratio of predicted seizures to the total number of seizures;
- Specificity/true negative rate (TNR): $\frac{TN}{TN + FP}$, the ratio of predicted non-seizures to the total number of non-seizures samples;
- AUC: area under the $\frac{TP}{TP + FN}$ and $\frac{FP}{FP + TN}$ curve, a probability value ranging from 0.5 to 1.

#### Model performance

After the NLP extraction of textual data and EEG mapping of EDF data, seizure signals are classified by using the six inputs described in Section .

The model parameters have been tested in multiple configurations. For RF, the total number of trees to be generated by the model has been set to 100. The model was also tested with 50 trees, where the model was found to be overfitting, and 150 trees, parameter that increased the execution time with minor improvements of the results. The minimum split of each tree was evaluated at the default value of 2 and with the parameter set to 5. However, since the dataset is large, when using the parameter set to 2 the model was underfitting, thus the minimum split of 5 performs better. To evaluate the quality of the split, we select the entropy measure as it is more sensitive to impurity and fit the data better than GINI index. For SVM, given non-linearly separable data, RBF kernel was selected. The regularization parameter (lambda) between 500 to 1200 and kernel coefficient (gamma) between 1e−10 to 10 were tested using grid-search. The model performs well with tight margin (high lambda value), but overfitting is detected when the influence of the support vectors have a large radius of the area (high gamma value). The regularization parameter of 1000 and kernel coefficient of 1e−9 were selected also taking in consideration of computational time. For MLP, the model was tested with 1 to 3 hidden layers and number of neurons at each layer from 16 to 1024. When trying to balance the fit of the model, one hidden layer with 256 neurons was found to perform the best. The other parameters were set to the default settings of the scikit-learn library. We omit the complete results, listing below the detailed summary of the model parameters used in the experiments:

- RF: A random forest classifier with entropy impurity, number of trees set to 100 and minimum number of sample split set to 5;
- SVM: A support vector machine classifier with RBF kernel, regularization parameter of 1000 and kernel coefficient of 1e−9.
- MLP: A multi-layer perceptron classifier with a single hidden layer with 256 neurons, ReLU activation function, Adam weight optimizer, regularization parameter of 1e−4.

The performance of sub-bands selection is listed in Table 6. The results of sub-zones selection are listed in Table 7. The data sample size and recording time duration (in seconds) of the six inputs are listed for three different electrode references in Table 9. In Table 8, the results of both sub-bands and sub-zones with six inputs of seizure type GNSZ are present as a representative sample. In this section, we present the results of the four seizure types GNSZ, TCSZ, FNSZ and CPSZ that are the most sizeable in the dataset. The results of seizure types ABSZ, MYSZ, TNSZ, SPSZ are listed in Appendix.

**Table 6:** “Selected Bands” performance metrics of seizure types GNSZ, TCSZ, FNSZ, CPSZ

			RF				SVM				MLP			
			ACC	AUC	TPR	TNR	ACC	AUC	TPR	TNR	ACC	AUC	TPR	TNR
GNSZ	01_tcp_ar	Selected_bands	0.936	0.972	0.669	0.99	0.933	0.942	0.692	0.982	0.88	0.914	0.757	0.906
		Background_bands	0.953	0.985	0.808	0.988	0.921	0.942	0.638	0.989	0.931	0.955	0.801	0.962
		Whole_frequency	0.97	0.993	0.894	0.989	0.947	0.969	0.791	0.985	0.951	0.975	0.86	0.973
	02_tcp_le	Selected_bands	0.937	0.987	0.933	0.94	0.935	0.985	0.915	0.953	0.871	0.923	0.894	0.849
		Background_bands	0.954	0.991	0.941	0.965	0.944	0.984	0.932	0.954	0.918	0.967	0.878	0.951
		Whole_frequency	0.972	0.996	0.969	0.974	0.955	0.989	0.947	0.962	0.937	0.983	0.923	0.949
	03_tcp_ar_a	Selected_bands	0.955	0.982	0.667	0.991	0.949	0.947	0.707	0.979	0.944	0.938	0.755	0.967
		Background_bands	0.96	0.982	0.672	0.995	0.946	0.939	0.602	0.988	0.937	0.939	0.557	0.983
		Whole_frequency	0.977	0.99	0.823	0.995	0.965	0.97	0.742	0.993	0.957	0.957	0.73	0.985
TCSZ	01_tcp_ar	Selected_bands	0.955	0.982	0.693	0.995	0.939	0.957	0.67	0.979	0.92	0.887	0.701	0.953
		Background_bands	0.961	0.989	0.807	0.99	0.951	0.973	0.779	0.983	0.91	0.948	0.861	0.921
		Whole_frequency	0.968	0.991	0.83	0.994	0.964	0.985	0.817	0.992	0.93	0.94	0.81	0.953
	02_tcp_le	Selected_bands	0.999	1	1	0.95	0.999	1	1	0.96	0.993	0.7	1	0.4
		Background_bands	0.983	0.998	0.983	0.984	0.981	0.996	0.983	0.979	0.96	0.969	0.971	0.944
		Whole_frequency	0.99	0.999	0.992	0.987	0.987	0.997	0.99	0.981	0.955	0.953	0.996	0.893
	03_tcp_ar_a	Selected_bands	0.976	0.997	0.8	0.994	0.971	0.986	0.856	0.986	0.958	0.984	0.884	0.966
		Background_bands	0.966	0.989	0.751	0.995	0.962	0.981	0.765	0.989	0.948	0.956	0.715	0.981
		Whole_frequency	0.99	0.998	0.93	0.999	0.989	0.997	0.937	0.996	0.944	0.982	0.907	0.95
FNSZ	01_tcp_ar	Selected_bands	0.858	0.904	0.527	0.973	0.819	0.816	0.383	0.97	0.791	0.708	0.317	0.955
		Background_bands	0.893	0.951	0.633	0.981	0.823	0.834	0.368	0.977	0.782	0.696	0.203	0.976
		Whole_frequency	0.937	0.982	0.79	0.987	0.869	0.9	0.551	0.975	0.837	0.797	0.466	0.962
	02_tcp_le	Selected_bands	0.898	0.949	0.561	0.989	0.887	0.913	0.575	0.972	0.84	0.869	0.629	0.897
		Background_bands	0.921	0.975	0.774	0.977	0.884	0.929	0.697	0.954	0.861	0.897	0.649	0.941
		Whole_frequency	0.956	0.99	0.877	0.986	0.913	0.954	0.773	0.965	0.897	0.941	0.741	0.956
	03_tcp_ar_a	Selected_bands	0.918	0.955	0.623	0.981	0.887	0.904	0.481	0.973	0.855	0.864	0.266	0.98
		Background_bands	0.891	0.949	0.647	0.968	0.848	0.887	0.535	0.948	0.829	0.829	0.405	0.964
		Whole_frequency	0.932	0.977	0.797	0.974	0.886	0.929	0.687	0.949	0.874	0.909	0.587	0.964
CPSZ	01_tcp_ar	Selected_bands	0.901	0.963	0.869	0.925	0.884	0.945	0.84	0.917	0.811	0.875	0.652	0.928
		Background_bands	0.935	0.982	0.88	0.967	0.897	0.954	0.807	0.949	0.878	0.925	0.771	0.94
		Whole_frequency	0.961	0.992	0.933	0.977	0.929	0.973	0.878	0.959	0.902	0.953	0.826	0.946
	02_tcp_le	Selected_bands	0.874	0.946	0.748	0.937	0.871	0.922	0.733	0.94	0.759	0.806	0.613	0.833
		Background_bands	0.933	0.982	0.88	0.962	0.919	0.966	0.86	0.952	0.842	0.913	0.818	0.855
		Whole_frequency	0.959	0.992	0.927	0.977	0.938	0.978	0.886	0.967	0.9	0.949	0.791	0.96
	03_tcp_ar_a	Selected_bands	0.871	0.933	0.604	0.97	0.839	0.883	0.61	0.924	0.734	0.753	0.596	0.786
		Background_bands	0.926	0.974	0.759	0.982	0.9	0.943	0.714	0.963	0.884	0.918	0.643	0.964
		Whole_frequency	0.968	0.993	0.908	0.988	0.944	0.977	0.851	0.975	0.929	0.967	0.803	0.972

**Table 7:** “Selected Zones” performance metrics of seizure type GNSZ, TCSZ, FNSZ, CPSZ

			RF				SVM				MLP			
			ACC	AUC	TPR	TNR	ACC	AUC	TPR	TNR	ACC	AUC	TPR	TNR
GNSZ	01_tcp_ar	Zoned_selected_bands	0.935	0.97	0.658	0.991	0.927	0.93	0.613	0.991	0.917	0.912	0.655	0.971
		Zoned_background_bands	0.948	0.983	0.8	0.984	0.92	0.945	0.65	0.985	0.923	0.949	0.791	0.955
		Zoned_whole_frequency	0.968	0.992	0.888	0.987	0.935	0.964	0.732	0.984	0.953	0.975	0.844	0.979
	02_tcp_le	Zoned_selected_bands	0.948	0.99	0.938	0.956	0.949	0.99	0.932	0.964	0.913	0.962	0.947	0.883
		Zoned_background_bands	0.959	0.993	0.955	0.962	0.945	0.985	0.931	0.956	0.93	0.979	0.898	0.956
		Zoned_whole_frequency	0.971	0.996	0.969	0.974	0.946	0.985	0.935	0.955	0.948	0.989	0.939	0.955
	03_tcp_ar_a	Zoned_selected_bands	0.958	0.983	0.673	0.994	0.941	0.935	0.632	0.979	0.936	0.919	0.688	0.967
		Zoned_background_bands	0.962	0.981	0.694	0.995	0.932	0.916	0.434	0.992	0.941	0.944	0.578	0.985
		Zoned_whole_frequency	0.972	0.987	0.802	0.993	0.952	0.954	0.609	0.994	0.962	0.972	0.744	0.988
TCSZ	01_tcp_ar	Zoned_selected_bands	0.952	0.98	0.689	0.992	0.938	0.961	0.667	0.979	0.909	0.877	0.705	0.94
		Zoned_background_bands	0.961	0.989	0.795	0.992	0.955	0.977	0.794	0.986	0.941	0.93	0.765	0.974
		Zoned_whole_frequency	0.967	0.99	0.832	0.992	0.955	0.978	0.766	0.991	0.937	0.944	0.774	0.967
	02_tcp_le	Zoned_selected_bands	0.999	0.979	1	0.96	0.999	1	1	0.96	0.997	0.979	0.997	0.96
		Zoned_background_bands	0.98	0.998	0.979	0.983	0.98	0.996	0.981	0.978	0.961	0.974	0.972	0.944
		Zoned_whole_frequency	0.989	0.999	0.99	0.987	0.985	0.998	0.984	0.987	0.98	0.988	0.984	0.974
	03_tcp_ar_a	Zoned_selected_bands	0.978	0.997	0.83	0.995	0.982	0.994	0.931	0.988	0.965	0.952	0.797	0.986
		Zoned_background_bands	0.975	0.991	0.829	0.996	0.969	0.976	0.839	0.987	0.937	0.938	0.757	0.962
		Zoned_whole_frequency	0.98	0.991	0.865	0.996	0.976	0.985	0.833	0.996	0.935	0.969	0.894	0.94
FNSZ	01_tcp_ar	Zoned_selected_bands	0.86	0.908	0.516	0.98	0.799	0.779	0.273	0.982	0.782	0.723	0.231	0.973
		Zoned_background_bands	0.893	0.947	0.636	0.979	0.822	0.826	0.360	0.978	0.818	0.783	0.419	0.952
		Zoned_whole_frequency	0.925	0.979	0.801	0.980	0.862	0.887	0.530	0.973	0.860	0.862	0.550	0.965
	02_tcp_le	Zoned_selected_bands	0.912	0.963	0.656	0.982	0.897	0.934	0.652	0.964	0.867	0.876	0.601	0.939
		Zoned_background_bands	0.922	0.973	0.784	0.974	0.886	0.931	0.704	0.955	0.88	0.925	0.689	0.952
		Zoned_whole_frequency	0.956	0.99	0.887	0.983	0.914	0.96	0.767	0.969	0.931	0.974	0.841	0.965
	03_tcp_ar_a	Zoned_selected_bands	0.912	0.953	0.592	0.98	0.869	0.885	0.37	0.976	0.847	0.781	0.206	0.984
		Zoned_background_bands	0.893	0.949	0.663	0.966	0.82	0.862	0.37	0.962	0.798	0.763	0.225	0.979
		Zoned_whole_frequency	0.921	0.97	0.783	0.964	0.863	0.913	0.581	0.952	0.892	0.936	0.692	0.956
CPSZ	01_tcp_ar	Zoned_selected_bands	0.91	0.97	0.903	0.915	0.888	0.949	0.862	0.907	0.849	0.926	0.747	0.925
		Zoned_background_bands	0.927	0.978	0.876	0.956	0.866	0.934	0.757	0.929	0.865	0.924	0.72	0.948
		Zoned_whole_frequency	0.955	0.991	0.923	0.974	0.919	0.97	0.845	0.963	0.916	0.967	0.824	0.969
	02_tcp_le	Zoned_selected_bands	0.914	0.972	0.844	0.949	0.912	0.959	0.823	0.957	0.83	0.878	0.753	0.869
		Zoned_background_bands	0.926	0.978	0.877	0.953	0.891	0.951	0.8	0.941	0.839	0.904	0.751	0.888
		Zoned_whole_frequency	0.959	0.992	0.929	0.976	0.93	0.973	0.869	0.964	0.897	0.961	0.911	0.89
	03_tcp_ar_a	Zoned_selected_bands	0.901	0.959	0.724	0.967	0.87	0.908	0.655	0.95	0.82	0.772	0.558	0.918
		Zoned_background_bands	0.936	0.979	0.813	0.978	0.902	0.938	0.708	0.967	0.9	0.937	0.694	0.968
		Zoned_whole_frequency	0.959	0.99	0.889	0.983	0.929	0.964	0.778	0.98	0.935	0.973	0.813	0.976

**Table 8:** Performance metrics of GNSZ with “sub-bands” and “sub-zones” selection

			RF				SVM				MLP			
			ACC	AUC	TPR	TNR	ACC	AUC	TPR	TNR	ACC	AUC	TPR	TNR
	01_tcp_ar	Zoned_selected_bands	0.935	0.97	0.658	0.991	0.927	0.93	0.613	0.991	0.917	0.912	0.655	0.971
		Selected_bands	0.936	0.972	0.669	0.99	0.933	0.942	0.692	0.982	0.88	0.914	0.757	0.906
		Zoned_background_bands	0.948	0.983	0.8	0.984	0.92	0.945	0.65	0.985	0.923	0.949	0.791	0.955
		Background_bands	0.953	0.985	0.808	0.988	0.921	0.942	0.638	0.989	0.931	0.955	0.801	0.962
		Zoned_whole_frequency	0.968	0.992	0.888	0.987	0.935	0.964	0.732	0.984	0.953	0.975	0.844	0.979
		Whole_frequency	0.97	0.993	0.894	0.989	0.947	0.969	0.791	0.985	0.951	0.975	0.86	0.973
	02_tcp_le	Zoned_selected_bands	0.948	0.99	0.938	0.956	0.949	0.99	0.932	0.964	0.913	0.962	0.947	0.883
		Selected_bands	0.937	0.987	0.933	0.94	0.935	0.985	0.915	0.953	0.871	0.923	0.894	0.849
GNSZ		Zoned_background_bands	0.959	0.993	0.955	0.962	0.945	0.985	0.931	0.956	0.93	0.979	0.898	0.956
		Background_bands	0.954	0.991	0.941	0.965	0.944	0.984	0.932	0.954	0.918	0.967	0.878	0.951
		Zoned_whole_frequency	0.971	0.996	0.969	0.974	0.946	0.985	0.935	0.955	0.948	0.989	0.939	0.955
		Whole_frequency	0.972	0.996	0.969	0.974	0.955	0.989	0.947	0.962	0.937	0.983	0.923	0.949
	03_tcp_ar_a	Zoned_selected_bands	0.958	0.983	0.673	0.994	0.941	0.935	0.632	0.979	0.936	0.919	0.688	0.967
		Selected_bands	0.955	0.982	0.667	0.991	0.949	0.947	0.707	0.979	0.944	0.938	0.755	0.967
		Zoned_background_bands	0.962	0.981	0.694	0.995	0.932	0.916	0.434	0.992	0.941	0.944	0.578	0.985
		Background_bands	0.96	0.982	0.672	0.995	0.946	0.939	0.602	0.988	0.937	0.939	0.557	0.983
		Zoned_whole_frequency	0.972	0.987	0.802	0.993	0.952	0.954	0.609	0.994	0.962	0.972	0.744	0.988
		Whole_frequency	0.977	0.99	0.823	0.995	0.965	0.97	0.742	0.993	0.957	0.957	0.73	0.985

**Table 9:** Size and recording time duration of “sub-bands” and “sub-zones” selection of seizure type GNSZ, TCSZ, FNSZ, CPSZ

		GNSZ		TCSZ		FNSZ		CPSZ	
		Size	Time	Size	Time	Size	Time	Size	Time
01_tcp_ar	Zoned_selected_bands	7148	26079	6632	13923	76560	335837	5560	47925
	Selected_bands	12232	26079	9180	13923	179460	335837	17692	47925
	Zoned_background_bands	29492	26079	16988	13923	337700	335837	36548	47925
	Background_bands	66880	26079	36576	13923	895984	335837	123936	47925
	Zoned_whole_frequency	295264	26079	134316	13923	2562332	335837	331404	47925
	Whole_frequency	490224	26079	234024	13923	5098632	335837	726264	47925
02_tcp_le	Zoned_selected_bands	1000	33004	500	5495	13000	58959	2500	20576
	Selected_bands	4500	33004	1000	5495	33500	58959	9500	20576
	Zoned_background_bands	21500	33004	3000	5495	50500	58959	14500	20576
	Background_bands	56000	33004	10000	5495	120000	58959	44000	20576
	Zoned_whole_frequency	125500	33004	18000	5495	309500	58959	99000	20576
	Whole_frequency	294000	33004	52500	5495	630000	58959	231000	20576
03_tcp_ar_a	Zoned_selected_bands	3712	34970	0	3778	6720	95778	3584	45787
	Selected_bands	28320	34970	800	3778	70688	95778	23168	45787
	Zoned_background_bands	18912	34970	800	3778	89920	95778	23872	45787
	Background_bands	74816	34970	6400	3778	284480	95778	129152	45787
	Zoned_whole_frequency	226848	34970	23200	3778	899616	95778	442336	45787
	Whole_frequency	559968	34970	60800	3778	2191840	95778	1071296	45787

### Ablation study

We performed an ablation study for the pre-defined electrodes in the zone list when classifying the seizures. More specifically, only five electrodes (A1, A2, CZ, C3, C4) are used for 01_tcp_ar and 02_tcp_le, and three electrodes (CZ, C3, C4) are used for 03_tcp_ar_a to conduct the experiments. The results of the study are shown in Table 10.

The objective for ablation study is threefold. Firstly, to evaluate the effectiveness of the zone selection by comparing the results with selected zones performance as shown in Table 6. Secondly, to assess the classification results by the essential zones of seizures as reported in previous studies [12, 13]. Thirdly, to provide the ground electrodes data for patients recording that does not have a specific description of zones in the clinical report. The pre-defined electrodes are consistent across all seizure types. The results can be compared both horizontally across seizures and vertically within seizures. By observing from Table 10, it is clear all four major seizure types have no data in set_zoned_selected_bands. Further, for 03_tcp_ar_a, both selected bands and background bands have no data samples. Distinguishing the same columns in Table 10 and Table 6, it is possible to recognize the potential of selected electrodes besides the pre-defined ones. The whole frequency data ablation results are promising, reveal the power of the essential electrodes, and confirm the results when compared to previous studies.

**Table 10:** Performance metrics of ablation study for seizure types GNSZ, TCSZ, FNSZ, CPSZ

			RF				SVM				MLP			
			ACC	AUC	TPR	TNR	ACC	AUC	TPR	TNR	ACC	AUC	TPR	TNR
GNSZ	01_tcp_ar	Set_zoned_selected_bands	0	0	0	0	0	0	0	0	0	0	0	0
		Set_zoned_background_bands	0.916	0.961	0.675	0.979	0.89	0.92	0.573	0.972	0.847	0.886	0.772	0.866
		Set_zoned_whole_frequency	0.963	0.99	0.865	0.987	0.94	0.961	0.755	0.984	0.948	0.974	0.869	0.968
	02_tcp_le	Set_zoned_selected_bands	0	0	0	0	0	0	0	0	0	0	0	0
		Set_zoned_background_bands	0.927	0.981	0.906	0.945	0.925	0.974	0.896	0.949	0.901	0.957	0.859	0.934
		Set_zoned_whole_frequency	0.959	0.993	0.952	0.966	0.946	0.984	0.94	0.951	0.915	0.973	0.852	0.966
	03_tcp_ar_a	Set_zoned_selected_bands	0	0	0	0	0	0	0	0	0	0	0	0
		Set_zoned_background_bands	0	0	0	0	0	0	0	0	0	0	0	0
		Set_zoned_whole_frequency	0.968	0.987	0.738	0.996	0.949	0.941	0.602	0.992	0.949	0.94	0.636	0.987
TCSZ	01_tcp_ar	Set_zoned_selected_bands	0	0	0	0	0	0	0	0	0	0	0	0
		Set_zoned_background_bands	0.957	0.987	0.824	0.989	0.958	0.971	0.869	0.98	0.93	0.923	0.835	0.953
		Set_zoned_whole_frequency	0.968	0.992	0.847	0.991	0.956	0.98	0.827	0.98	0.916	0.931	0.811	0.935
	02_tcp_le	Set_zoned_selected_bands	0	0	0	0	0	0	0	0	0	0	0	0
		Set_zoned_background_bands	0.878	0.96	0.805	0.988	0.865	0.94	0.802	0.961	0.832	0.825	0.801	0.873
		Set_zoned_whole_frequency	0.986	0.998	0.986	0.986	0.987	0.998	0.989	0.983	0.976	0.988	0.972	0.981
	03_tcp_ar_a	Set_zoned_selected_bands	0	0	0	0	0	0	0	0	0	0	0	0
		Set_zoned_background_bands	0	0	0	0	0	0	0	0	0	0	0	0
		Set_zoned_whole_frequency	0.978	0.993	0.846	0.996	0.976	0.986	0.872	0.99	0.952	0.949	0.724	0.984
FNSZ	01_tcp_ar	Set_zoned_selected_bands	0	0	0	0	0	0	0	0	0	0	0	0
		Set_zoned_background_bands	0.83	0.887	0.608	0.933	0.792	0.838	0.508	0.924	0.791	0.813	0.56	0.899
		Set_zoned_whole_frequency	0.907	0.961	0.686	0.982	0.828	0.847	0.391	0.975	0.797	0.674	0.261	0.977
	02_tcp_le	Set_zoned_selected_bands	0	0	0	0	0	0	0	0	0	0	0	0
		Set_zoned_background_bands	0.863	0.921	0.602	0.962	0.831	0.882	0.503	0.956	0.8	0.821	0.408	0.948
		Set_zoned_whole_frequency	0.927	0.977	0.797	0.977	0.882	0.935	0.669	0.962	0.874	0.925	0.675	0.95
	03_tcp_ar_a	Set_zoned_selected_bands	0	0	0	0	0	0	0	0	0	0	0	0
		Set_zoned_background_bands	0	0	0	0	0	0	0	0	0	0	0	0
		Set_zoned_whole_frequency	0.903	0.959	0.7	0.968	0.851	0.891	0.522	0.955	0.82	0.789	0.406	0.951
CPSZ	01_tcp_ar	Set_zoned_selected_bands	0	0	0	0	0	0	0	0	0	0	0	0
		Set_zoned_background_bands	0.869	0.941	0.722	0.946	0.835	0.906	0.659	0.927	0.81	0.856	0.566	0.937
		Set_zoned_whole_frequency	0.947	0.987	0.912	0.967	0.911	0.961	0.833	0.955	0.876	0.935	0.745	0.952
	02_tcp_le	Set_zoned_selected_bands	0	0	0	0	0	0	0	0	0	0	0	0
		Set_zoned_background_bands	0.88	0.95	0.769	0.941	0.855	0.92	0.767	0.904	0.728	0.824	0.736	0.729
		Set_zoned_whole_frequency	0.937	0.984	0.892	0.963	0.923	0.969	0.857	0.959	0.895	0.949	0.838	0.927
	03_tcp_ar_a	Set_zoned_selected_bands	0	0	0	0	0	0	0	0	0	0	0	0
		Set_zoned_background_bands	0	0	0	0	0	0	0	0	0	0	0	0
		Set_zoned_whole_frequency	0.94	0.981	0.817	0.98	0.901	0.94	0.702	0.967	0.892	0.924	0.675	0.964

## Discussion

The classification experiment in the study evaluated two main type (sub-bands and sub-zones) with six pre-defined inputs (Zoned Selected Bands, Selected Bands, Zoned Background Bands, Background Bands, Zoned Whole Frequency, Whole Frequency) to answer the three research questions.

The extracted keyword aggregation presented in Table 5 shows the importance of the recording EEG signals in frontal, temporal and central zones of the brain cortex. Temporal lobe electrodes often mentioned in clinical reports, and the term prevail in seizure type GNSZ, TNSZ, CPSZ, CPSZ. Besides temporal lobe, electrodes placed at central or frontal are carrying significant functions with a large proportion that may affect seizure classification performance. Also, the occurrence of the keywords in frequency bands are listed out in 4. Theta is predominant in GNSZ, FNSZ and CPSZ, while beta is often observed and mentioned in seizure type TNSZ and TCSZ when compared to other frequency bands, delta does not outweigh other bands for seizure classification. The two aggregations also show that pre-frontal brain cortex and alpha activities are often neglected in expert’s reports.

Table 6 with inputs 2, 4, and 6 aims to answer *RQ1*, namely, how selection of $\alpha , \beta , \theta , \delta , \gamma$ influence the classification. Table 7 with inputs 1 3 5 tries to answer *RQ2*, how selecting brain zones in addition to frequency bands affects the seizure classification.

With certain frequency filtered out the performance metrics drops in almost all the experiments, as it may be expected, with the exception of some minor points where it improved. However, for seizure classification problem, the measurement of accuracy and specificity does not play a vital role. On the other hand, important measures are the sensitivity refers to the model’s ability to detect the real seizure as positive, and AUC indicates the model’s ability to distinguish of ictal/inter-ictal. In general, the results of the Random Forest classifier outperforms all the other classification algorithms here considered. Although the sensitivity and AUC in sub-bands selection deviate from using whole frequency, the deviation of background frequency bands selection are acceptable given that the time of execution is reduced. This proved the effectiveness of selecting background frequency bands as a potential classification approach. Channel selection results are more consistent, bringing to a considerately more plausible approach. Comparing the three groups of sub-bands (input 1 & 2, input 3 & 4, input 5 & 6), with specific electrodes filtered out, the results achieve better results than using all the channels.

**Fig. 6:**
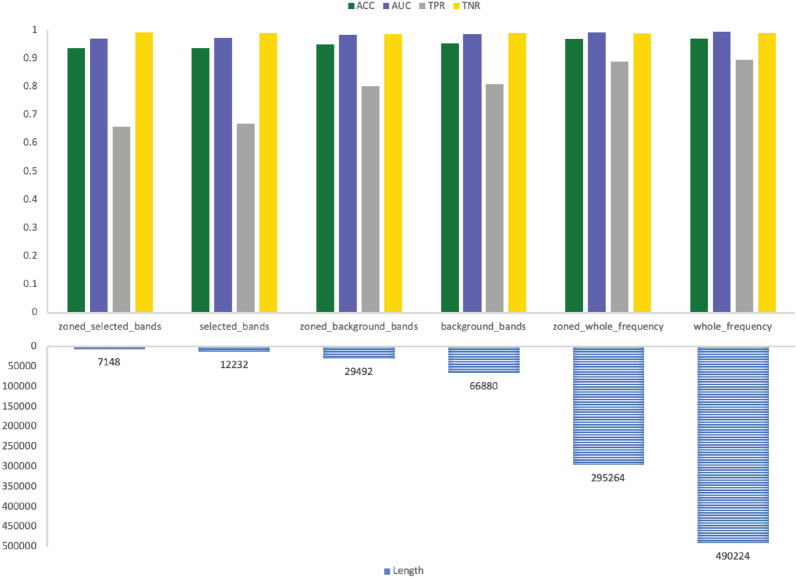
GNSZ 01_tcp_ar seizure classification with Random Forest using the six data selection methodologies

**Fig. 7:**
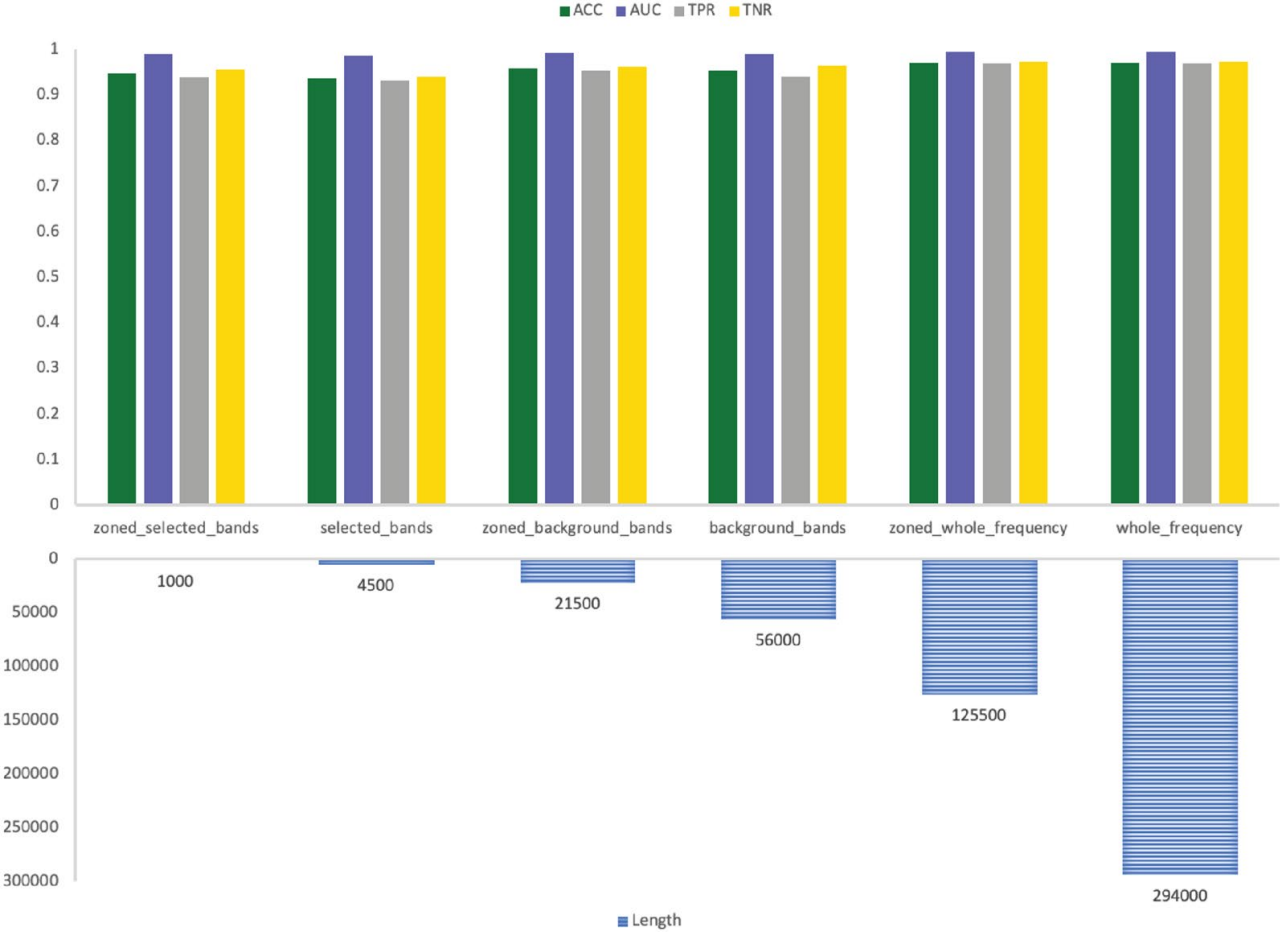
GNSZ 02_tcp_le seizure classification with Random Forest using the six data selection methodologies

**Fig. 8:**
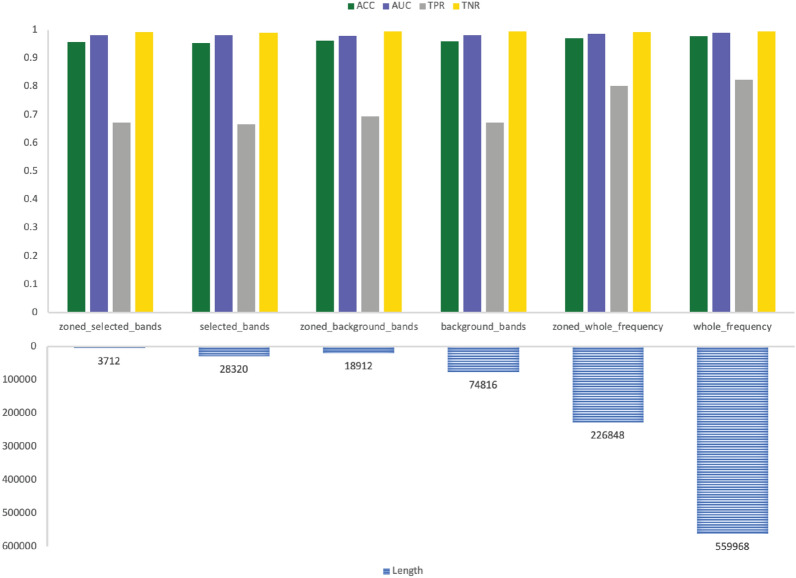
GNSZ 03_tcp_ar_a seizure classification with Random Forest using the six data selection methodologies

**Fig. 9:**
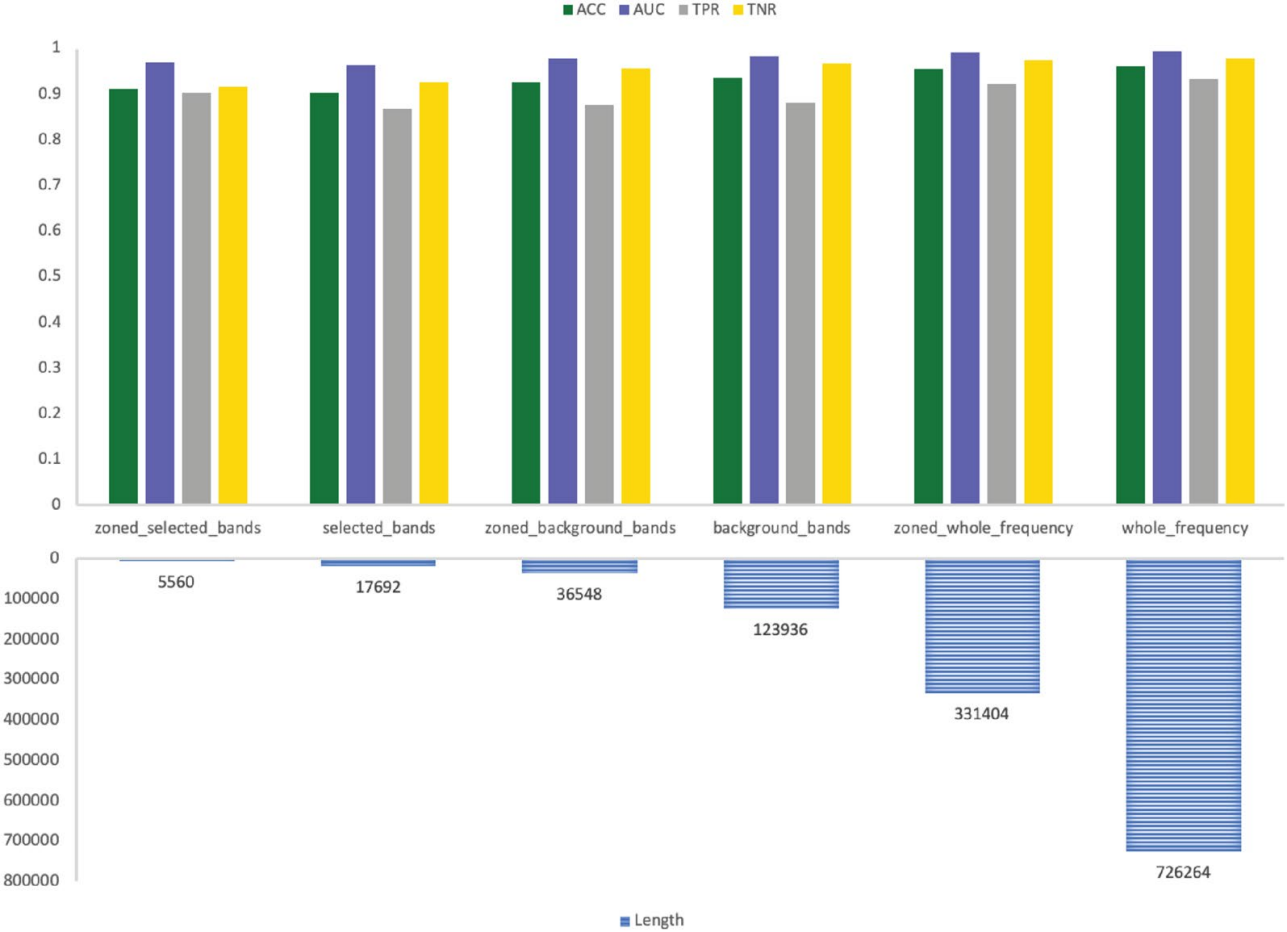
CPSZ 01_tcp_ar seizure classification with Random Forest using the six data selection methodologies

**Fig. 10:**
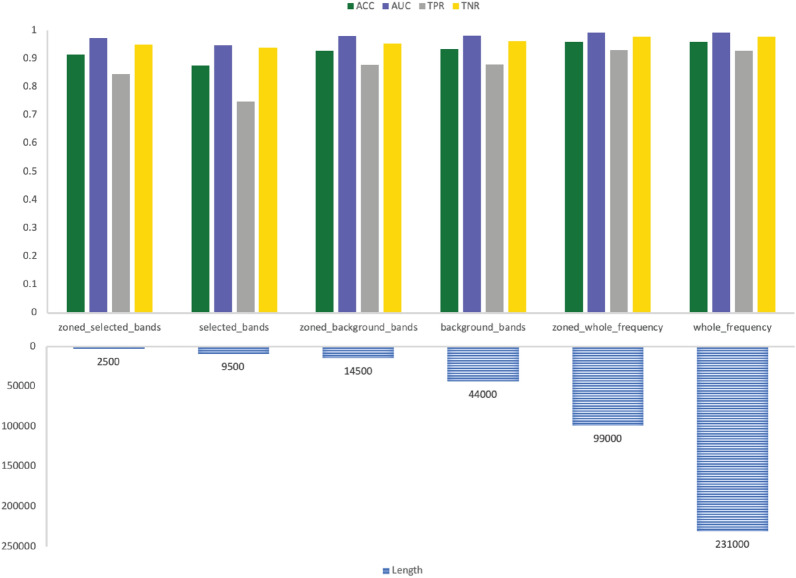
CPSZ 02_tcp_le seizure classification with Random Forest using the six data selection methodologies

**Fig. 11:**
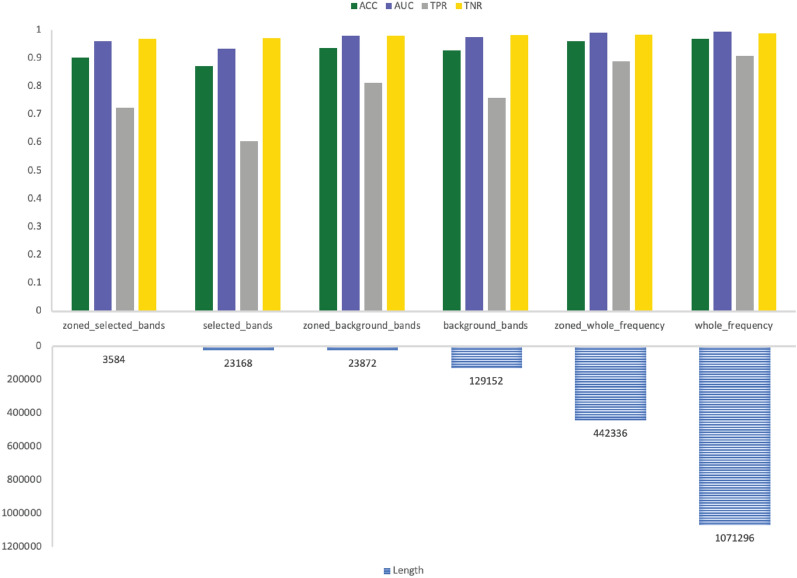
CPSZ 03_tcp_ar_a seizure classification with Random Forest using the six data selection methodologies

The results are more comprehensive when looking at both sub-bands and sub-zones selection together. The performance metrics of GNSZ, taken as a representative sample, are shown in Table 8 with the size of the six inputs listed in Table 9. The RF results are visualized in Fig. 6 for the reference electrodes 01_tcp_ar, in Fig. 7 for reference electrodes 02_tcp_le, and in Fig. 8 for reference electrodes 03_tcp_ar_a. From the charts is visually evident that the quantity of data is reduced significantly with each input configuration filters out information. The performance matrix for Zoned Whole Frequency and Whole Frequency only deviated around 0.01 for all the measures. The Zoned Whole Frequency approach achieves excellent classification results using nearly half of the quantity of data when compared to the Whole Frequency. The results can be replicated for other seizure types with similar performances. Additional information is reported in the results table and, for seizure CPSZ, is visually represented in Figs. 9, 10, and 11.

## Conclusions and future works

In this work, we introduced a novel natural language processing approach to predict seizure using EEG data. The approach is based on the efficient selection of frequency bands and scalp EEG electrodes to reduce the computational time and quantity of data needed for the seizure classification.

By analyzing the patients’ clinical reports, we integrated the prior knowledge into the classifier-building process, mimicking the authentic thinking process of experts’ opinion for seizure diagnosis with EEG data. In particular, we classify seizure ictal/inter-ictal phases with three types of frequency band inputs: 1) the whole frequency range provided in data corpus; 2) the background frequency EEG bands $(\alpha , \beta , \theta , \delta , \gamma )$, and 3) the selected background bands based on individual’s clinical reports extracted by Natural Language Processing (NLP). Together with the frequency band selection, we additionally used the scalp EEG electrodes reduction by NLP analysis.

The experiment results show that by integrating prior knowledge from experts to build individualized seizure classification models, interesting results can be achieved. Using prior knowledge for the selection of EEG electrodes and frequency bands influence the quantity of data that the classification model analyzed. This led to a more efficient classification of the input data, achieving excellent results with the selection of electrodes. Mixed results have been achieved when selecting the frequency bands.

Using the proposed approach, we introduced a novel methodology for patient-specific seizure detection method using frequency bands and selected electrodes. The algorithm is computationally efficient, compared to the whole band classification. Results show that using the proposed approach may lead to more efficient implementations of the seizure classifier to be executed on power-efficient devices for long-lasting real-time detection of seizures.

In future works, we will further explore the classification of EEG using more advanced NLP techniques on clinical reports, to extract additional information on the thinking process of medics when analyzing EEG data.

## Data Availability

The dataset is open to public with no charge by Temple University Hospital. Link to the dataset: https://isip.piconepress.com/projects/tuh_eeg/html/downloads.shtml.

## Appendix

Tables 11 and 12 report the top 50 keywords extracted from clinical report using the Natural Language Processing techniques described in narrative.

**Table 11:** Top 50 keywords from clinical reports for GNSZ, ABSZ, MYSZ, and TNSZ seizure types

Rank	GNSZ		ABSZ		MYSZ		TNSZ	
	Word	Frequency	Word	Frequency	Word	Frequency	Word	Frequency
1	Eeg	0.365	Activity	0.329	Activity	0.451	Seizures	0.316
2	Seizures	0.238	Spike	0.241	Wave	0.27	Eeg	0.251
3	Patient	0.238	Wave	0.221	Spike	0.24	Seen	0.235
4	Activity	0.235	Child	0.211	Eeg	0.18	Patient	0.235
5	Left	0.209	Eeg	0.201	Clinical	0.18	Spike	0.162
6	Clinical	0.178	Clinical	0.177	10	0.15	Recording	0.154
7	Right	0.168	Seizure	0.165	Patient	0.15	Seizure	0.154
8	Seizure	0.146	Slow	0.157	Record	0.15	Wave	0.154
9	Record	0.14	Record	0.157	Myoclonus	0.143	Activity	0.145
10	Background	0.122	Hz	0.152	Myoclonic	0.126	Tonic	0.131
11	Recording	0.114	Generalized	0.137	Pattern	0.12	Multiple	0.129
12	Sharp	0.114	Photic	0.126	Background	0.12	Generalized	0.129
13	Performed	0.112	Stimulation	0.126	fFndings	0.112	Clinical	0.129
14	10	0.112	Burst	0.123	Predominant	0.1	Detection	0.125
15	Slowing	0.112	Seconds	0.118	Stimulation	0.1	Diffusely	0.121
16	Temporal	0.104	10	0.118	Photic	0.1	Performed	0.121
17	Continuous	0.097	Amplitude	0.118	2012	0.095	20	0.121
18	Pattern	0.095	Drowsy	0.117	Abnormal	0.09	Diffuse	0.117
19	20	0.093	Seen	0.115	Performed	0.09	Seconds	0.113
20	Hemisphere	0.093	High	0.113	Hz	0.09	Beta	0.108
21	Monitoring	0.093	Background	0.113	Slow	0.09	Left	0.105
22	Seen	0.093	Left	0.113	1	0.084	Software	0.102
23	History	0.09	Hyperventilation	0.11	Vanco	0.075	Addition	0.099
24	Noted	0.09	Predominant	0.104	dnr1	0.075	Right	0.097
25	Video	0.09	Performed	0.103	Prognostic	0.075	Record	0.097

Rank	GNSZ		ABSZ		MYSZ		TNSZ	
	Word	Frequency	Word	Frequency	Word	Frequency	Word	Frequency
26	Region	0.089	Right	0.103	vdrf	0.075	Normal	0.091
27	Focal	0.086	Seizures	0.103	Frontally	0.075	p3	0.091
28	Waves	0.085	Discharge	0.099	Alpha	0.075	Waves	0.089
29	Theta	0.081	Frontally	0.098	30	0.067	Discharges	0.081
30	Description	0.08	Sleep	0.098	Excess	0.067	Epilepsy	0.081
31	Frequency	0.079	Bursts	0.088	Rhythm	0.067	Cerebral	0.081
32	Amplitude	0.076	History	0.083	Epilepsy	0.067	Amplitude	0.081
33	Sleep	0.074	Patient	0.083	Post	0.067	Slow	0.081
34	Medications	0.074	Second	0.077	Jerking	0.067	History	0.081
35	Abnormal	0.073	Slowing	0.071	Status	0.067	Hz	0.081
36	Delta	0.072	Absence	0.07	Morphine	0.063	Abnormal	0.081
37	Epileptiform	0.072	Poly	0.07	Traumatic	0.063	10	0.072
38	Status	0.072	UV	0.07	Ready	0.063	Background	0.072
39	Ekg	0.071	Occurs	0.069	Percocet	0.063	Electrode	0.072
40	Posterior	0.071	Duration	0.068	dvt	0.063	Onset	0.072
41	Electrode	0.07	Posterior	0.068	History	0.06	Procedures	0.071
42	Diffuse	0.069	Video	0.068	Electrode	0.06	Activating	0.071
43	Frontal	0.068	Frontal	0.068	High	0.06	Frequent	0.071
44	Wave	0.067	Epilepsy	0.066	ekg	0.06	Followed	0.064
45	Standard	0.065	Focal	0.064	Introduction	0.06	Sleep	0.064
46	Slow	0.065	Correlation	0.064	Impression	0.06	Etiology	0.064
47	Anterior	0.064	Staring	0.062	Old	0.06	Sharp	0.063
48	Rhythmic	0.064	Identified	0.061	Noted	0.06	Appears	0.063
49	Introduction	0.061	Features	0.061	Left	0.06	Opens	0.06
50	Hz	0.06	Discharges	0.06	Focal	0.06	Consistent	0.06

**Table 12:** Top 50 keywords from clinical reports for TCSZ, FNSZ, SPSZ, and CPSZ seizure types

Rank	TCSZ		FNSZ		SPSZ		CPSZ	
	Word	Frequency	Word	Frequency	Word	Frequency	Word	Frequency
1	Eeg	0.291	Eeg	0.306	Seizures	0.495	Seizures	0.318
2	Right	0.281	left	0.274	Activity	0.256	Eeg	0.311
3	Seizure	0.266	Right	0.253	Eeg	0.238	patient	0.29
4	Left	0.264	Activity	0.252	Left	0.238	Activity	0.24
5	Patient	0.231	Seizures	0.235	Hemisphere	0.19	Seizure	0.238
6	Activity	0.228	Patient	0.217	Patient	0.179	Left	0.228
7	Seizures	0.185	Seizure	0.197	Clinical	0.179	Right	0.22
8	Tonic	0.128	Clinical	0.158	Right	0.145	Clinical	0.166
9	Sleep	0.115	Slowing	0.132	Beta	0.114	Partial	0.165
10	Clonic	0.111	Temporal	0.13	Record	0.11	Complex	0.153
11	Hemisphere	0.11	Region	0.126	Section	0.107	Record	0.141
12	Clinical	0.109	Record	0.124	Sleep	0.102	Temporal	0.13
13	Record	0.109	Continuous	0.119	Temporal	0.085	10	0.112
14	Region	0.105	Background	0.107	Monitoring	0.085	Region	0.107
15	Background	0.105	10	0.107	10	0.085	Slowing	0.104
16	10	0.105	Sharp	0.106	Rhythmic	0.085	Sharp	0.09
17	Seen	0.099	Focal	0.106	Delta	0.085	Frequency	0.089
18	Sharp	0.099	Hemisphere	0.104	History	0.076	Video	0.086
19	Recording	0.096	Performed	0.099	Pattern	0.076	Background	0.083
20	Arm	0.095	Posterior	0.096	Video	0.076	Performed	0.083
21	Waves	0.092	Frequency	0.096	Performed	0.076	Hemisphere	0.08
22	Temporal	0.088	Waves	0.094	Recording	0.076	Status	0.078
23	Performed	0.086	Monitoring	0.086	0	0.076	20	0.075
24	20	0.082	Recording	0.085	Bursts	0.076	Continuous	0.074
25	History	0.079	Video	0.084	Central	0.076	Recording	0.071

Rank	TCSZ		FNSZ		SPSZ		CPSZ	
	Word	Frequency	Word	Frequency	Word	Frequency	Word	Frequency
26	Amplitude	0.076	20	0.083	Occur	0.074	Monitoring	0.071
27	Slow	0.076	Diffuse	0.081	Shaking	0.074	Noted	0.07
28	Time	0.074	Noted	0.081	Region	0.068	Waves	0.068
29	Slowing	0.073	Electrodes	0.08	Seconds	0.068	Description	0.067
30	Anterior	0.07	Delta	0.079	Theta	0.068	Rhythmic	0.067
31	Rhythm	0.07	Status	0.077	20	0.068	Seen	0.066
32	Occipital	0.069	History	0.077	Noted	0.068	Electrode	0.064
33	Pattern	0.069	Description	0.076	Introduction	0.068	Epileptiform	0.063
34	Generalized	0.069	Pattern	0.076	Buzz	0.067	Posterior	0.063
35	Hz	0.069	Theta	0.076	Fast	0.066	Using	0.063
36	Artifact	0.066	Seen	0.075	30	0.066	Pattern	0.061
37	Electrode	0.066	Epileptiform	0.073	Characterized	0.066	History	0.061
38	Onset	0.062	Amplitude	0.073	Polyspike	0.059	ekg	0.06
39	Focal	0.062	Moderate	0.071	Description	0.059	Introduction	0.06
40	Ictal	0.06	Occipital	0.071	Impression	0.059	Impression	0.059
41	Electrodes	0.06	Abnormal	0.067	Duration	0.059	Theta	0.058
42	Wave	0.059	Medications	0.067	bpm	0.059	Placement	0.056
43	Monitoring	0.057	Sleep	0.063	Medications	0.059	Abnormal	0.056
44	Delta	0.056	Epilepticus	0.063	Epileptiform	0.057	Focal	0.056
45	Abnormal	0.056	ekg	0.063	Seizure	0.057	Slow	0.056
46	Posterior	0.056	Polymorphic	0.061	Service	0.054	Repetitive	0.055
47	Muscle	0.055	Rhythmic	0.06	Sections	0.053	Epilepticus	0.055
48	Epileptiform	0.055	Standard	0.06	24	0.053	Channel	0.055
49	Dominant	0.055	Electrode	0.057	Multiple	0.051	Medications	0.054
50	Voltage	0.053	Electrographic	0.057	hr	0.051	Digital	0.054

Performance metrics for seizure types ABSZ, MYSZ, TNSZ, SPSZ are shown in Table 13.

**Table 13:** Performance metric of selected zones of seizure type ABSZ, MYSZ, TNSZ, SPSZ

			RF				SVM				MLP			
			ACC	AUC	TPR	TNR	ACC	AUC	TPR	TNR	ACC	AUC	TPR	TNR
ABSZ	01_tcp_ar	Zoned_selected_bands	0.997	0.996	0.816	0.999	0.998	0.998	0.883	1	0.998	0.973	0.883	0.999
		Zoned_background_bands	0.999	1	0.933	1	0.999	0.999	0.933	1	0.999	1	0.933	1
		Zoned_whole_frequency	0.998	0.999	0.883	1	0.998	0.999	0.883	1	0.995	0.959	0.833	0.997
	02_tcp_le	Zoned_selected_bands	0.994	0.994	0.944	0.996	0.992	0.982	0.887	0.996	0.989	0.957	0.848	0.995
		Zoned_background_bands	0.992	0.995	0.883	0.996	0.991	0.99	0.868	0.996	0.986	0.961	0.894	0.989
		Zoned_whole_frequency	0.994	0.996	0.903	0.997	0.994	0.993	0.901	0.997	0.99	0.962	0.836	0.996
MYSZ	01_tcp_ar	Zoned_selected_bands	0.993	0.998	0.55	1	0.994	0.998	0.916	0.995	0.994	0.997	0.85	0.997
		Zoned_background_bands	0.995	0.997	0.6	1	0.991	0.994	0.716	0.995	0.991	0.994	0.533	0.996
		Zoned_whole_frequency	0.994	0.998	0.7	0.997	0.997	0.999	0.916	0.999	0.993	0.997	0.666	0.998
	02_tcp_le	Zoned_selected_bands	0.997	0.93	1	0.869	0.996	0.973	0.998	0.869	0.994	0.933	0.996	0.869
		Zoned_background_bands	0.997	0.957	1	0.869	0.996	0.912	0.998	0.869	0.997	0.934	1	0.869
		Zoned_whole_frequency	0.997	0.977	1	0.869	0.998	0.967	1	0.919	0.995	0.88	1	0.72
TNSZ	01_tcp_ar	Zoned_selected_bands	0.942	0.885	0.275	0.996	0.934	0.849	0.343	0.982	0.843	0.733	0.416	0.878
		Zoned_background_bands	0.945	0.901	0.305	0.997	0.944	0.891	0.303	0.997	0.899	0.794	0.402	0.939
		Zoned_whole_frequency	0.949	0.912	0.353	0.998	0.947	0.917	0.359	0.995	0.881	0.705	0.426	0.918
SPSZ	01_tcp_ar	zoned_selected_bands	0.905	0.951	0.6	0.984	0.852	0.859	0.425	0.963	0.868	0.87	0.604	0.933
		Zoned_background_bands	0.933	0.972	0.726	0.986	0.914	0.946	0.685	0.973	0.888	0.919	0.8	0.91
		Zoned_whole_frequency	0.945	0.981	0.801	0.983	0.917	0.954	0.678	0.979	0.922	0.949	0.802	0.952

**Table 14:** Performance metric of selected bands of seizure type ABSZ, MYSZ, TNSZ, SPSZ

			RF				SVM				MLP			
			ACC	AUC	TPR	TNR	ACC	AUC	TPR	TNR	ACC	AUC	TPR	TNR
ABSZ	01_tcp_ar	Selected_bands	0.995	0.994	0.593	1	0.998	0.999	0.883	0.999	0.996	0.973	0.843	0.998
		Background_bands	0.998	0.999	0.883	1	0.999	0.999	0.933	1	0.998	0.974	0.909	0.999
		Whole_frequency	0.997	0.965	0.833	0.999	0.998	0.999	0.833	1	0.996	0.998	0.843	0.998
	02_tcp_le	Selected_bands	0.994	0.997	0.92	0.996	0.994	0.996	0.923	0.996	0.988	0.958	0.895	0.992
		Background_bands	0.992	0.994	0.889	0.996	0.991	0.985	0.866	0.996	0.982	0.951	0.848	0.987
		Whole_frequency	0.993	0.995	0.898	0.997	0.993	0.988	0.896	0.997	0.99	0.959	0.832	0.996
MYSZ	01_tcp_ar	Selected_bands	0.991	0.995	0.419	1	0.992	0.992	0.593	0.998	0.991	0.97	0.673	0.996
		Background_bands	0.993	0.998	0.616	0.999	0.995	0.998	0.773	0.999	0.993	0.964	0.606	0.998
		Whole_frequency	0.993	0.998	0.733	0.997	0.997	0.999	0.893	0.999	0.995	0.999	0.736	0.998
	02_tcp_le	Selected_bands	0.997	0.925	1	0.766	0.997	0.893	0.999	0.866	0.993	0.932	0.996	0.766
		Background_bands	0.997	0.999	1	0.766	0.996	0.998	0.998	0.866	0.984	0.596	0.993	0.2
		Whole_frequency	0.997	0.999	1	0.766	0.997	1	1	0.766	0.993	0.75	1	0.5
TNSZ	01_tcp_ar	Selected_bands	0.942	0.876	0.279	0.996	0.938	0.838	0.313	0.989	0.887	0.753	0.395	0.928
		Background_bands	0.944	0.894	0.305	0.997	0.944	0.885	0.312	0.996	0.892	0.826	0.501	0.924
		Whole_frequency	0.948	0.912	0.343	0.998	0.956	0.913	0.484	0.995	0.866	0.79	0.489	0.897
SPSZ	01_tcp_ar	Selected_bands	0.912	0.948	0.618	0.988	0.878	0.898	0.579	0.955	0.865	0.878	0.651	0.92
		Background_bands	0.946	0.98	0.782	0.988	0.925	0.955	0.776	0.964	0.907	0.919	0.637	0.975
		Whole_frequency	0.956	0.987	0.823	0.991	0.928	0.964	0.747	0.975	0.904	0.956	0.819	0.928

Table 14 shows the performance metrics of selected bands for seizure type ABSZ, MYSZ, TNSZ, SPSZ.

Table 15 shows data sample size and recording time duration in seconds of seizure type ABSZ, MYSZ, TNSZ, SPSZ.

**Table 15:** Size and recording time duration of “sub-bands” and “sub-zones” selection of seizure type ABSZ, MYSZ, TNSZ, SPSZ

		ABSZ		MYSZ		TNSZ		SPSZ	
		Size	Time	Size	Time	Size	Time	Size	Time
01_tcp_ar	zoned_selected_bands	0	1530	0	1465	14324	15833	512	3977
	Selected_bands	500	1530	500	1465	14324	15833	2560	3977
	Zoned_background_bands	500	1530	500	1465	28136	15833	3072	3977
	Background_bands	2000	1530	2000	1465	57296	15833	10240	3977
	Zoned_whole_frequency	4000	1530	4000	1465	183688	15833	19968	3977
	Whole_frequency	10500	1530	10500	1465	300804	15833	53760	3977
02_tcp_le	zoned_selected_bands	3000	22537	0	1305	0	0	0	0
	Selected_bands	4500	22537	1000	1305	0	0	0	0
	Zoned_background_bands	15500	22537	0	1305	0	0	0	0
	Background_bands	38000	22537	4000	1305	0	0	0	0
	Zoned_whole_frequency	96000	22537	3000	1305	0	0	0	0
	Whole_frequency	199500	22537	21000	1305	0	0	0	0

Table 16 shows performance metric of ablation study for seizure type ABSZ, MYSZ, TNSZ, SPSZ.

**Table 16:** Performance metric of ablation study of seizure type ABSZ, MYSZ, TNSZ, SPSZ

			RF				SVM				MLP			
			ACC	AUC	TPR	TNR	ACC	AUC	TPR	TNR	ACC	AUC	TPR	TNR
ABSZ	01_tcp_ar	Set_zoned_selected_bands	0	0	0	0	0	0	0	0	0	0	0	0
		Set_zoned_background_bands	0.997	0.996	0.816	0.999	0.998	0.998	0.883	1	0.998	0.973	0.883	0.999
		Set_zoned_whole_frequency	0.997	0.999	0.833	0.999	0.998	0.999	0.883	0.999	0.998	0.998	0.933	0.998
	02_tcp_le	Set_zoned_selected_bands	0	0	0	0	0	0	0	0	0	0	0	0
		Set_zoned_background_bands	0.991	0.993	0.873	0.996	0.99	0.978	0.846	0.995	0.98	0.929	0.834	0.985
		Set_zoned_whole_frequency	0.991	0.993	0.862	0.996	0.991	0.982	0.851	0.997	0.985	0.903	0.66	0.997
MYSZ	01_tcp_ar	Set_zoned_selected_bands	0	0	0	0	0	0	0	0	0	0	0	0
		Set_zoned_background_bands	0.993	0.998	0.55	1	0.994	0.998	0.916	0.995	0.994	0.997	0.85	0.997
		Set_zoned_whole_frequency	0.995	0.999	0.666	1	0.997	1	0.966	0.998	0.994	0.997	0.766	0.997
	02_tcp_le	Set_zoned_selected_bands	0	0	0	0	0	0	0	0	0	0	0	0
		Set_zoned_background_bands	0.997	0.945	1	0.766	0.996	0.995	0.999	0.766	0.985	0.712	0.993	0.4
		Set_zoned_whole_frequency	0.997	0.999	1	0.766	0.998	0.999	1	0.866	0.997	0.883	1	0.766
TNSZ	01_tcp_ar	Set_zoned_selected_bands	0	0	0	0	0	0	0	0	0	0	0	0
		Set_zoned_background_bands	0.983	0.966	0.694	0.996	0.983	0.92	0.724	0.994	0.95	0.54	0.123	0.982
		Set_zoned_whole_frequency	0.949	0.926	0.364	0.997	0.948	0.908	0.389	0.994	0.909	0.808	0.401	0.95
SPSZ	01_tcp_ar	Set_zoned_selected_bands	0	0	0	0	0	0	0	0	0	0	0	0
		Set_zoned_background_bands	0	0	0	0	0	0	0	0	0	0	0	0
		Set_zoned_whole_frequency	0.945	0.985	0.767	0.99	0.926	0.963	0.765	0.967	0.911	0.943	0.751	0.955

## Abbreviations

EEG: Electroencephalography
NLP: Natural Language Processing
FFT: Fast Fourier Transform
STFT: Short-time Fourier Transform
AR: Average Reference
LE: Linked Ears Reference
REST: Reference Electrode Standardization Technique
IED: Inter-ictal Epileptiform Discharges
GNSZ: Generalized Seizure
ABSZ: Absence Seizure
MYSZ: Myoclonic Seizure
ATSZ: Atonic Seizure
TNSZ: Tonic Seizure
CNSZ: Clonic Seizure
TCSZ: Tonic–Clonic Seizure
FNSZ: Focal Seizure
SPSZ: Simple Partial Seizure
CPSZ: Complex pPartial Seizure
ILAE: International League Against Epilepsy
SOZ: Seizure-Onset Zone
ECoG: Intracranial ElectroCOrticoGraphic recordings
PDFs: Probability Density Functions
ICU: Intensive Care Unit
TUH: Temple University Hospital
TUSZ: Temple University Hospital Seizure Corpus
EDF: European Data Format
TXT: Text format
EMU: Epilepsy Monitoring Unit
STD: Standard Deviation
TF-IDF: Term frequency-inverse document frequency
RF: Random Forest
SVM: Support Vector Machines
MLP: Multi-Layer Perceptron
DT: Decision Tree
QP: Quadratic Programming
RBF: Radial-Basis Kernel
ReLU: Rectifier Linear Unit
ACC: Accuracy
AUC: Area Under Curve
TPR: Sensitivity
TNR: Specificity
TP: True Positive
FP: False Positive
TN: True Negative
FN: False Negative
